# Genomic survey sequencing, development and characterization of single- and multi-locus genomic SSR markers of *Elymus sibiricus* L

**DOI:** 10.1186/s12870-020-02770-0

**Published:** 2021-01-06

**Authors:** Yi Xiong, Xiong Lei, Shiqie Bai, Yanli Xiong, Wenhui Liu, Wendan Wu, Qingqing Yu, Zhixiao Dong, Jian Yang, Xiao Ma

**Affiliations:** 1 Department of Grassland Science, Animal Science and Technology College, Sichuan Agricultural University, Chengdu, 611130 China; 2 Sichuan Academy of Grassland Science, Chengdu, 611731 China; 3 Key Laboratory of Superior Forage Germplasm in the Qinghai-Tibetan Plateau, Qinghai Academy of Animal Science and Veterinary Medicine, Xining, 81108 China; 4 Sichuan Pratacultural Technology Research and Extension Center, Chengdu, 610041 China

**Keywords:** Genomic survey sequencing, *Elymus sibiricus* L., Marker development, Single-locus SSR, Genetic diversity study

## Abstract

**Background:**

Siberian wildrye (*Elymus sibiricus* L.) attracts considerable interest for grassland establishment and pasture recovery in the Qinghai-Tibet Plateau (QTP) due to its excellence in strong stress tolerance, high nutritional value and ease to cultivate. However, the lack of genomic information of *E. sibiricus* hampers its genetics study and breeding process.

**Results:**

In this study, we performed a genome survey and developed a set of SSR markers for *E. sibiricus* based on Next-generation sequencing (NGS). We generated 469.17 Gb clean sequence which is 58.64× of the 6.86 Gb estimated genome size. We assembled a draft genome of 4.34 Gb which has 73.23% repetitive elements, a heterozygosity ratio of 0.01% and GC content of 45.68%. Based on the gnomic sequences we identified 67,833 SSR loci and from which four hundred were randomly selected to develop markers. Finally, 30 markers exhibited polymorphism between accessions and ten were identified as single-locus SSR. These newly developed markers along with previously reported 30 ones were applied to analyze genetic polymorphism among 27 wild *E. sibiricus* accessions. We found that single-locus SSRs are superior to multi-loci SSRs in effectiveness.

**Conclusions:**

This study provided insights into further whole genome sequencing of *E. sibiricus* in strategy selection. The novel developed SSR markers will facilitate genetics study and breeding for *Elymus* species.

**Supplementary Information:**

The online version contains supplementary material available at 10.1186/s12870-020-02770-0.

## Background

As the largest genus in the tribe Triticeae of family Poaceae, *Elymus* L. contains approximately 150 species occurring in most temperate regions of the world [1]. Given its excellent resistance to drought, cold and disease, the *Elymus* species can provide important gene pool for improvement and breeding of related cereal crops [2]. Siberian wildrye (*Elymus sibiricus* L.), a model species of *Elymus* genus, is a perennial, cool-season and self-pollinated forage grass [1]. It widely spreads in northern Eurasia, and is especially used in the Qinghai-Tibet Plateau (QTP) for grassland establishment and restoration because of its strong adaptability, cold-resistance, high nutritional value, good palatability and ease for cultivation [3, 4]. Up to now, increasing attention has been paid to its germplasm characterization, intraspecific genetic diversity, phylogenic evolution and linkage map construction [1, 4, 5]. However, the lack of genomic sequences hinders the genetics study and breeding progress of this species compared to other Triticeae cereals such as wheat and barley.

*E. sibiricus* has an allotetraploid genome (StStHH, 2n = 4x = 28). The mean nuclear DNA content (C-value) of *E. sibiricus* was determined by flow cytometry (FCM) as being 2C = 16.61 pg, approximately twice as large as the possible diploid progenitors from genus *Pseudoroegneria* (StSt) and *Hordeum* (HH) [6]. This complexity and huge genome size pose a great challenge to whole genome sequencing of *E. sibiricus*. Genome survey using next-generation sequencing (NGS) with a given uniform sequencing depth is an alternative and cost-effective strategy in obtaining genome size, heterozygosity, GC and repeat contents. Furthermore, development of molecular markers based on genome survey sequencing and in silico analysis has become a practical tool for genetic study [7].

Among the various types of molecular markers, SSRs (simple sequence repeats) have many advantages including high polymorphism, codominant heredity, good reproducibility and extensive distribution in the genome [8]. These properties have proven to be of great interest for diverse genetic studies including genetic map and fingerprint construction, genetic diversity characterization, and molecular marker assisted breeding, etc. SSR markers can be developed using homology searches from either genomic libraries or transcriptome sequences and expressed sequence tags (ESTs) databases. Often, G-SSR (genomic DNA-derived SSRs) markers are considered to possess higher polymorphism than EST-SSR markers (EST-derived SSRs) due to conservation of the transcribed portions of genome. The ongoing development of next-generation sequencing (NGS) techniques e.g. genomic survey sequencing has economically allowed to access large amounts of genomic data, and can further identify genomic SSR loci by in silico searching SSR motifs in massive scaffold datasets [9–12].

In general, SSRs are believed to be locus-specific i.e. single-locus markers, therefore, only one or two bands (homozygotes or heterozygotes) was expectedly amplified with a single SSR primer pair. However, complex banding pattern (multiple loci) in addition to the expected ones (single-locus) was frequently obtained by a single SSR primer pair [13]. This may be explained by the fact that each of the markers targeted more than one homoeolocus due to the large genome size (especially for polyploid origin) as well as the high proportion of repetitive DNA in the genome of higher plants [9, 13, 14]. The multi-loci SSR markers brings many difficulties in the precise identification of genomic loci containing the specific genes of interest. For example, in the practical application of some polyploid species, the amplified products of multi-loci SSR by gel electrophoresis may be from multiple loci of multiple genomes, leading to problems such as error in genotyping and inaccurate calculation of diversity index [15]. On the contrary, compared to multi-loci SSRs, single-locus SSRs primers target a unique location in the genome and could provide more reliable scoring of genotypes in genetic study and breeding programs.

In this study genomic survey sequencing was applied in a *E. sibiricus* cultivar ‘Chuancao No.2’ using Illumina Hiseq X-ten platform. The first draft genome of *E. sibiricus* was constructed and some single-locus SSRs and multi-loci SSRs were developed based on genomic survey data. Furthermore, genetic diversity and structure of 27 wild *E. sibiricus* accessions were characterized using these new SSR markers plus previously published ones. Effectiveness of those markers was also compared and evaluated.

## Results

### Genome sequencing and characterization

Fourteen 270-bp libraries with pair-end reads of *E. sibiricus* were constructed and sequenced. The randomly selected 10,000 pairs of reads were then analyzed by BLAST using the NCBI databases. The BLAST result showed that *Triticum aestivum* and *Hordeum vulgare*, as the closely-related species to *Elymus*, were the best matching species in all libraries. In addition, reads of each library aligned with chloroplast genome of *E. sibiricus* showed lower than 5% matching rate, which indicated that the libraries were established without contamination (data not shown).

**Fig. 1:**
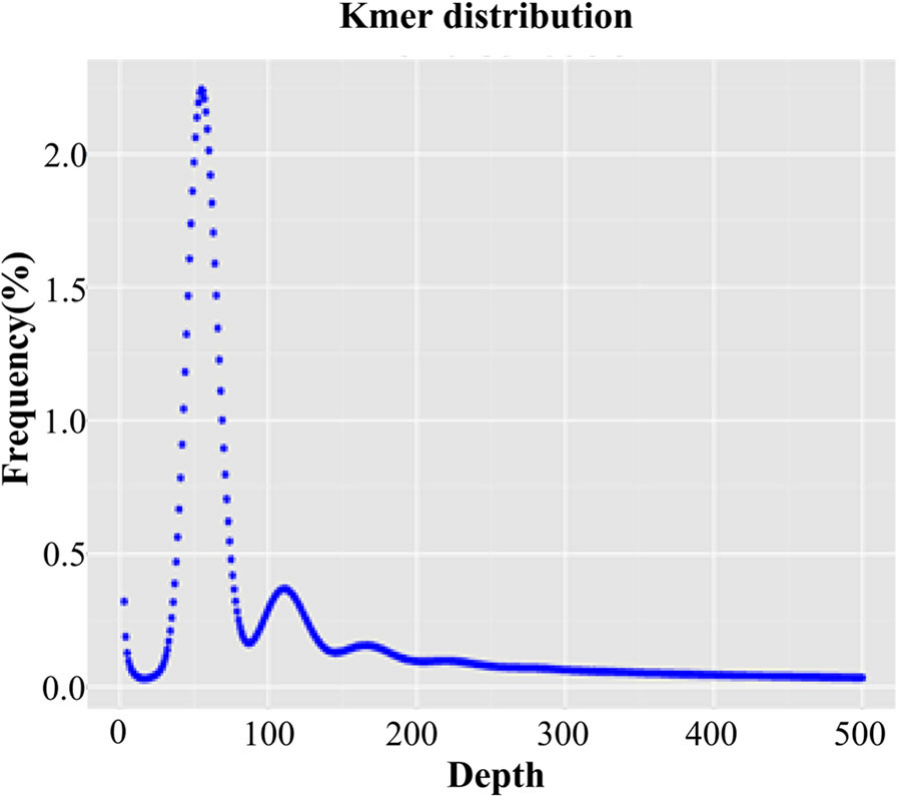
K-mer (k = 25) frequency distribution. The x-axis is depth and the y-axis represent the frequency at that depth. The genome size was calculated following ‘genome size = K-mer num/peak depth’. The two subpeaks were caused by certain repeats rate in the genome of *E. sibiricus*

From fourteen libraries with 270 bp insertion size, totally 469.17 Gb of clean data were produced using an Illumina HiSeq X-ten platform. The estimated genome size of *E. sibiricus* was approximately 6.86 Gb with a total sequencing depth of 58.64-fold (Table 1). High quality scores of the filtered sequences were calculated, and the percentages of Q20 and Q30 (the sequencing error rate 1 and 0.1% respectively) was greater than 95.81 and 90.50% respectively, indicating the high accuracy of the sequencing process (Table 1). Then all of the clean data were subjected to 25-mer (k = 25) frequency distribution analysis. It showed that the peak value of the k-mer depth distribution emerged at 54 (Fig. 1). The heterozygosity rate and the proportion of repeat sequence was calculated to be 0.01 and 73.23% respectively according to the k-mer curve distribution.

**Table 1:** Summary of the fourteen libraries with 270 bp short-inserts

Library	Total length (Gb) of high-quality reads	Depth (×)	Q20 (%)	Q30 (%)
270bp_1	34.85	5.08	97.26	93.35
270bp_2	32.56	4.74	97.24	93.30
270bp_3	31.22	4.55	97.17	93.17
270bp_4	34.77	5.07	97.2	93.24
270bp_5	27.88	4.06	97.68	94.25
270bp_6	35.98	5.24	97.67	94.22
270bp_7	35.44	5.16	97.69	94.26
270bp_8	38.09	5.55	97.95	94.81
270bp_9	32.92	4.8	97.7	94.27
270bp_10	35.76	5.21	97.62	94.11
270bp_11	32.02	4.66	97.53	93.92
270bp_12	36.92	5.38	97.81	94.52
270bp_13	31.16	4.54	**95.81**	**90.50**
270bp_14	29.60	4.31	95.91	90.70
Total	469.17	58.64	–	–

Depth (×), sequencing depth; Q20 (%), percentage of bases with sequencing error rate 1%; Q30 (%), percentage of bases with sequencing error rate 0.1%

**Fig. 2:**
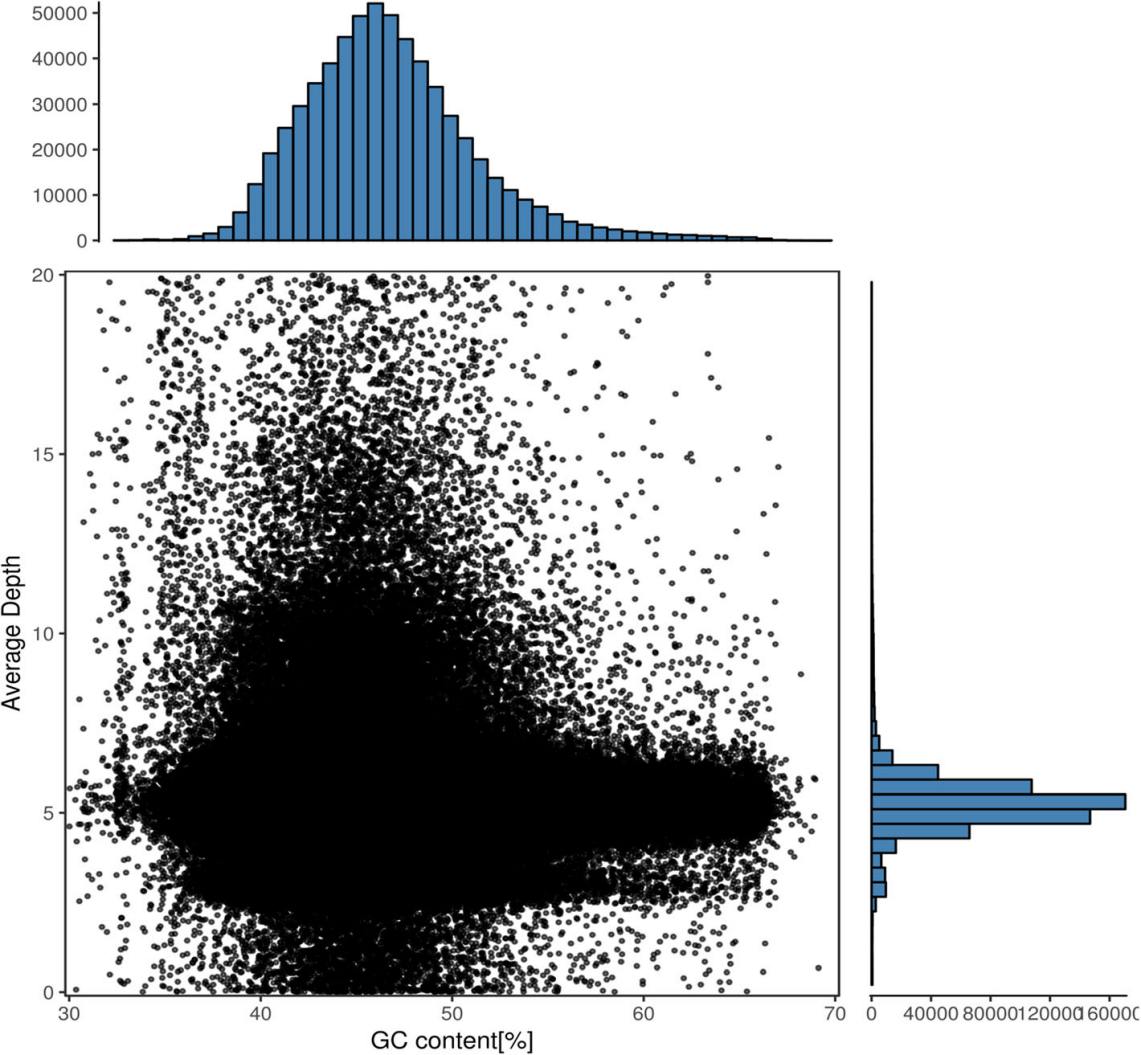
Guanine plus cytosine (GC) content and depth correlation analysis. The x-axis represents the GC content and the y-axis is the sequence depth

After de novo assembly using the SOAP de novo program, all of the clean reads produced a total of 4,841,088 contigs with an N50 length of 2510 bp, which were subsequently assembled into scaffolds (Table 2). Among all those contigs, 683,040 contigs were longer than 500 bp, 352,851 contigs were longer than 1 kb and 7536 contigs were longer than 10 kb. Scaffolds larger than 100 bp were selected for further analysis. Totally 4,763,904 scaffolds were generated with an N50 of 2648, and the number of scaffolds longer than 500 bp, 1 kb and 10 kb were 684,597, 352,659 and 8040, respectively (Table 2). The assembled draft genome was approximately 4.34 Gb, accounting for 63.27% of the estimated 6.86 Gb genome. The calculated GC content of the assembled genome was 45.68% (Table 2), that was consistent with the scatter plot graph built with scaffolds larger than 500 bp (Fig. 2). A total of 25,993 genes were annotated in the *E. sibiricus* draft genome with an average transcript length per gene of 2632.11 bp and an average coding sequence length of 737.36 bp. The predicted average exons number per gene was 4.72 and the average exon length per transcript was 311.32 bp (Table S1).

**Table 2:** Statistics of de novo assembly

	Contig		Scaffold		Genome	
	Size (bp)	Number	Size (bp)	Number	Size (bp)	Number
N50	2510		2648			
N90	306		338			
Total size	1,954,520,301		1,957,956,552		4,337,715,088	2,506,979
Total number		4,841,088		4,763,904		
Total number (≥500 bp)		683,040		684,597		
Total number (≥1 kb)		352,851		352,659		
Total number (≥10 kb)		7536		8040		
A					1,183,700,514	
T					1,152,964,970	
C					1,016,945,388	
G					1,033,545,446	
N					943,217	
Total (ACGT)					4,387,156,318	
GC content (ACGT)					45.68

Using RepeatMasker software [16], we annotated the repeat regions of the draft genome, in which the most abundant repetitive elements was retroelements (16.45%), followed by DNA transposons (3.26%). The most common element in retroelements and transposons were long terminal repeat (LTR, 15.90%) and Tc1-IS630-Pogo (0.25%), respectively (Table S2).

### Development of genome-wide SSR markers

**Fig. 3:**
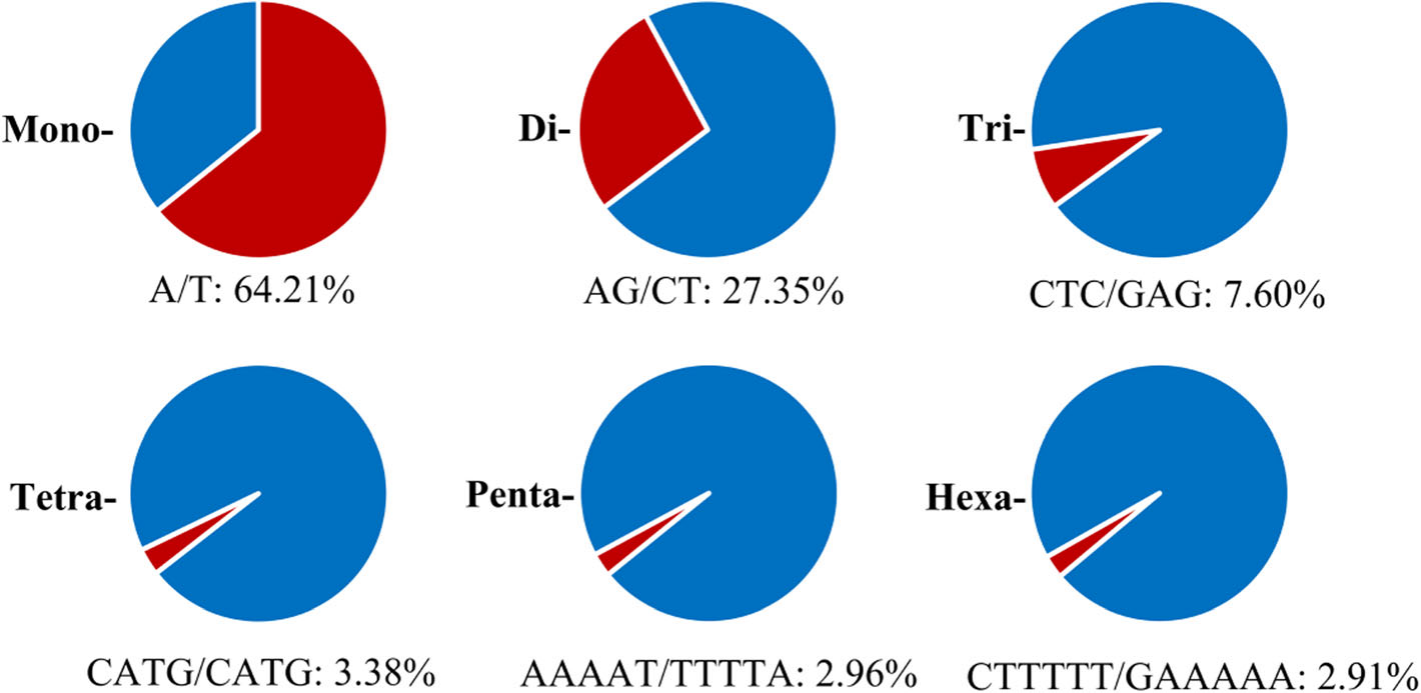
The most abundant motifs (red portion) corresponding to mono- to hexa-nucleotide repeats

**Fig. 4:**
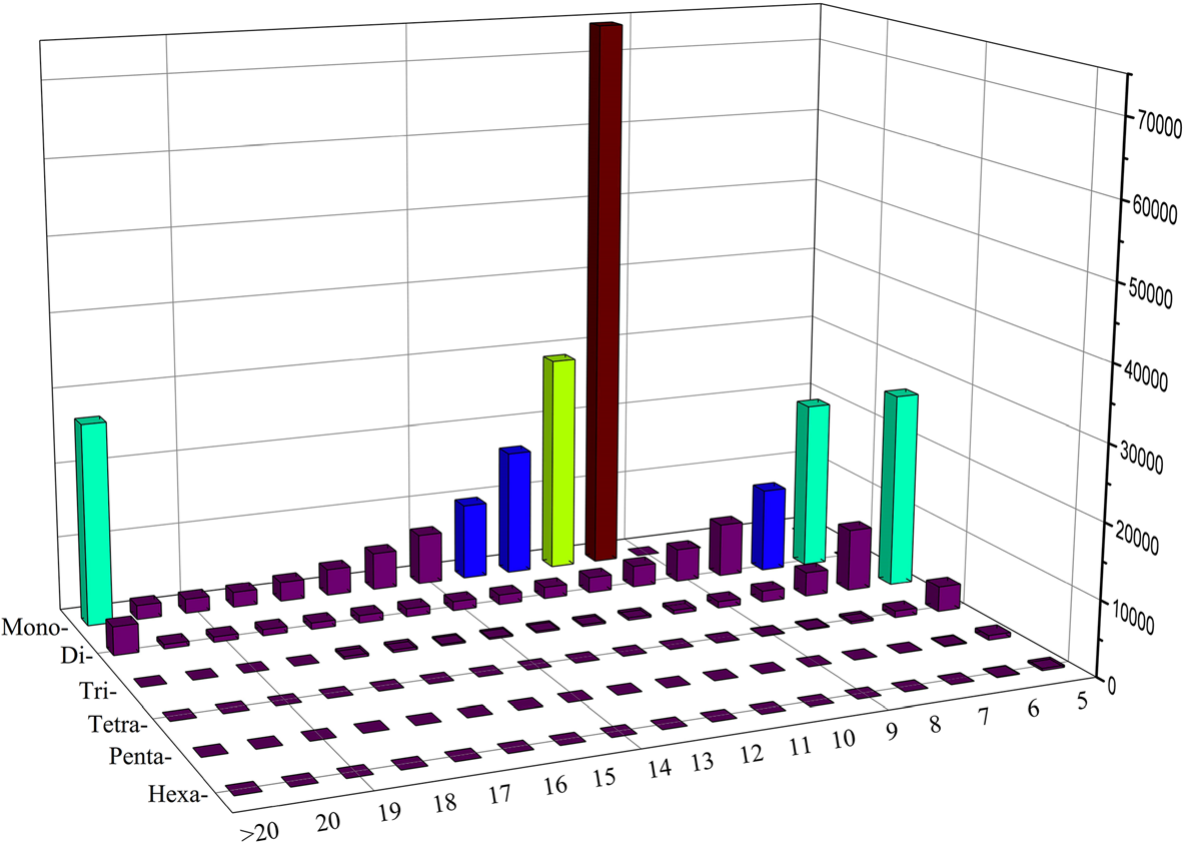
Motif frequency distributions of mono- to hexanucleotide motif types (y-axis) with different repeat numbers (from 5 to > 20, x-axis) in the de novo assembled genomic sequences of *E. sibiricus*. The z-axis represents the number of each type of motif

About 4.34 Gb genome sequences were searched for SSR loci and resulted in 315,446 SSRs from 507,162 (20.23%) scaffolds. 22,611 (0.90%) sequences contained a single-locus SSR which was not found on other scaffolds (Table S3). In these SSRs, the mono-, di- and tri-nucleotide motifs were the most enriched, which accounted for 61.81, 21.59 and 14.55% of the total identified SSRs, respectively (Table 3). Nucleotide composition characteristics indicated that A/T (64.21%), AG/CT (27.35%), CTC/GAG (7.6%), CATG/CATG (3.38%), AAAAT/TTTTA (2.96%) and CTTTTT/GAAAAA (2.91%) were the most abundant motifs corresponding to mono- to hexa-nucleotide repeats, respectively (Fig. 3). Generally, motif abundance decreased as the motif repeat number increased for each motif type (Fig. 4). The top three abundant motif repeat number were 10, 6 and 11, whose total number of SSR motifs were 77,930, 31,980 and 31,575 respectively (Table S4). Using Primer3 software [17], a total of 67,833 SSR primer pairs were designed for above-mentioned 22,611 single-locus SSR-containing sequences. In silico analysis was then used to verify the reliability and polymorphism of these primers by aligning the flanking sequences of them to the genome sequences. Four hundred SSR markers were randomly selected to amplify the genomic DNA of eight *E. sibiricus* accessions (Table S5) and 30 of them displayed polymorphisms and ten showed single-locus amplicons as expected (ESGA-SL) and 20 displayed multi-loci amplicons (ESGA-ML) (Table S6 and Fig. S1).

**Table 3:** Statistics of SSR motif

Motif	Number	Ratio	Accumulate Ratio
Mono-nucleotide	181,314	61.81%	61.81%
Di-nucleotide	63,342	21.59%	83.40%
Tri-nucleotide	42,695	14.55%	97.95%
Tetra-nucleotide	4857	1.66%	99.56%
Penta-nucleotide	777	0.26%	99.61%
Hexa-nucleotide	377	0.13%	100.00%

### Polymorphism of novel SSR markers in *E. sibiricus*

#### Marker polymorphism evaluation

A total of 60 SSR markers (30 ESGA, 15 ES and 15 ESGS, Table S6) were used to genotype 27 *E. sibiricus* accessions, which were collected from Mongolia (MG), eastern Qinghai-Tibet Plateau (QTP) and Siberia (SI) respectively. A total number of 29 alleles (Na) were produced by ten ESGA-SL markers, with an average of 2.9 (Table 4). The average polymorphism information content (PIC) value of all ESGA-SL markers was 0.391, among which the ESGA-SL-7 had the highest PIC value of 0.595. The mean value of observed heterozygosity (H_o_) calculated based on ESGA-SL markers was 0.480, among which ESGA-SL-10 had the highest H_o_ of 0.963.

**Table 4:** Genetic indexes of ESGA-SL markers

Primer ID	N_a_	PIC	H_o_
ESGA-SL-1	3	0.515	0.087
ESGA-SL-2	5	0.506	0.481
ESGA-SL-3	3	0.448	0.231
ESGA-SL-4	3	0.519	0.852
ESGA-SL-5	2	0.103	0.115
ESGA-SL-6	2	0.221	0.222
ESGA-SL-7	4	0.595	0.926
ESGA-SL-8	2	0.069	0.074
ESGA-SL-9	3	0.558	0.852
ESGA-SL-10	2	0.375	0.963
Total	29	/	/
Minimum	2	0.069	0.074
Maximum	4	0.595	0.963
Mean	2.9	0.391	0.480

*N*_*a*_ observed alleles number. *PIC* polymorphism information content. *H*_*o*_ observed heterozygosity

For multi-loci markers, 105, 97 and 67 polymorphic bands were amplified by ESGA-ML, ESGS and ES primers, and their corresponding average polymorphism percentage was 90.52, 82.49 and 73.84%, respectively (Table S7). Newly developed ESGA-ML markers had the highest average value of PIC (0.4059) and band informativeness (BI, 0.6494) compared to other multi-loci markers (ESGS and ES). Mann Whitney test [18] indicated that there was no significant difference between ESGA-SL and multi-loci markers in view of PIC values (Table S8). For MI, BI and Rp values, the significant difference (*P* < 0.01) was observed between transcriptome-developed ES marker and genomic-developed ESGA-ML and ESGS markers.

#### Cluster and STRUCTURE analysis

**Fig. 5:**
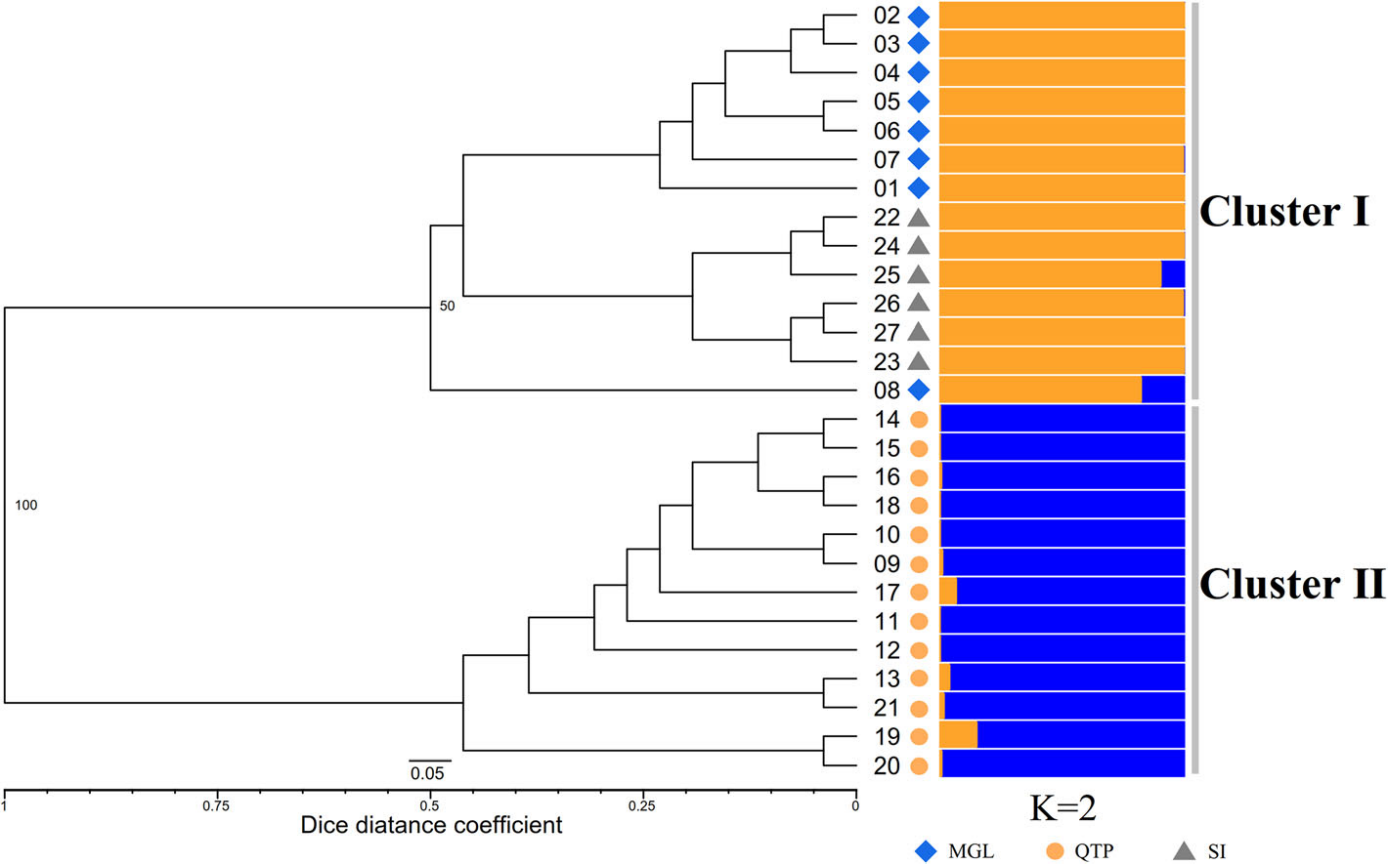
The UPGMA dendrogram and genetic structure of 27 studied *E. sibiricus* based on ESGA-SL markers. MGL, Mongolia; QTP, eastern Qinghai-Tibet Plateau; SI, Siberia

**Fig. 6:**
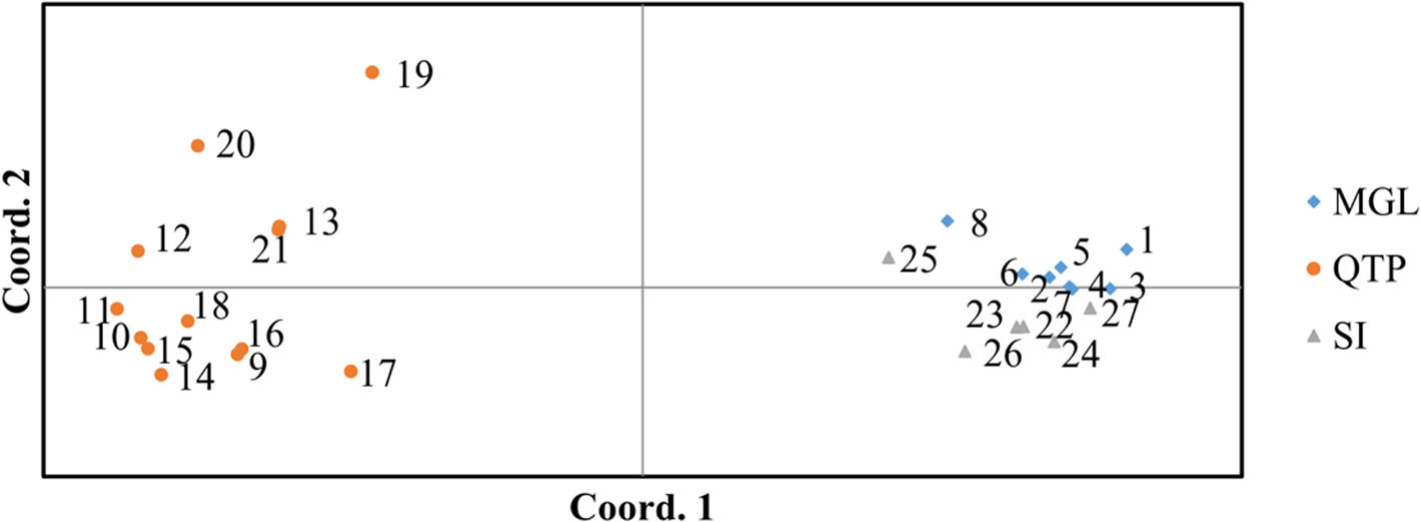
The PcoA analysis of 27 studied *E. sibiricus* based on ESGA-SL markers

Genetic memberships of the 27 tested *E. sibiricus* accessions based on ESGA-SL and ESGA-ML datasets were acquired via STRUCTURE 2.3.4 software (Fig. 5 and Fig. S2). The result revealed an optimal K value of 2 (K = 2), implying the tested accessions belonged to two potential genetic memberships, which was consistent with the UPGMA dendrogram and principal coordinates analysis (PCoA) using ESGA-SL markers. The 27 accessions could be divided into two clusters (Cluster I and Cluster II, Figs. 5 and 6). Cluster I included all the Mongolia (MG) and Siberia (SI) accessions, and Cluster II included all eastern QTP originated ones. Thus, the wild germplasms from different regions could be notably identified through the UPGMA and PCoA analysis, which revealed the powerful discriminability for wild accessions based on ESGA-SL markers. However, in spite of distinct characterization of tested accessions from different regions characterized via PCoA analysis based on ESGA-ML markers (Fig. S3), the topological structure of UMPGA dendrogram was ambiguous and couldn’t distinguish the 27 wild accessions clearly (Fig. S2). Furthermore, the similar result of UPGMA cluster and PCoA analysis based on ESGA-ML markers was also found in ESGS markers (Fig. S4 and Fig. S5). Besides, compared to genomic-developed markers, the transcriptome-developed ES markers could not explain their structure membership well (Fig. S6 and Fig. S7). This indicates that G-SSR markers has the superior discriminability than the EST-SSR markers for intraspecific diversity analysis.

#### The genetic diversity pattern of *E. sibiricus* based on different types of markers

Based on geographical origin, all tested wild accessions were divided into three geo-groups: MGL (Mongolia), SI (Siberia) and QTP (eastern Qinghai-Tibet Plateau). The observed heterozygosity (H_o_) of each geo-group based on the ESGA-SL dataset was changing from 0.467 to 0.492 (Table 5). In general, the values of N_a_, N_e_ (effective alleles number), I (Shannon information index), H_e_ (expected heterozygosity) and PP (Percentage of polymorphic loci) of each geo-group calculated by ESGA-SL marker were higher than that of ESGA-ML (Table 5). AMOVA analysis was carried out based on both the ESGA-SL and ESGA-ML markers and coefficient of genetic differentiation (F_st_) were calculated. The results showed that genetic variation of tested germplasms was mainly distributed among geo-groups with moderate genetic differentiation (F_st_ = 0.553 for ESGA-SL and F_st_ = 0.573 for ESGA-ML) (Table 6).

**Table 5:** Genetic diversity of studied *E. sibiricus* geo-groups based on ESGA-SL and ESGA-ML markers

Type	Geo-groups	N	N_a_	N_e_	I	H_o_	H_e_	PP
ESGA-SL	MGL	8	2.700	2.131	0.770	0.474	0.456	90.00%
	SI	6	2.000	1.758	0.514	0.467	0.326	60.00%
	QTP	13	2.800	1.971	0.741	0.492	0.438	100.00%
	Mean	–	2.500	1.953	0.675	0.478	0.407	83.33%
ESGA-ML	MGL	8	1.304	1.299	0.269	–	0.176	56.52%
	SI	6	1.183	1.280	0.247	–	0.164	47.83%
	QTP	13	1.478	1.352	0.304	–	0.203	61.74%
	Mean	–	1.322	1.310	0.273	–	0.181	55.36%

*N* Population size; *N*_*a*_ alleles number; *N*_*e*_ effective alleles number; *I* Shannon information index; *H*_*o*_ observed heterozygosity; *H*_*e*_ expected heterozygosity; *PP* Percentage of polymorphic loci; *MGL* Mongolia; *SI* Siberia; *QTP* eastern Qinghai-Tibet Plateau

**Table 6:** Genetic variation of *E. sibiricus* geo-groups

Type	Source	df	SS	PMV (%)	F_st_	N_m_	*P*-value
ESGA-SL	Among geo-groups	2	74.960	55.28%	0.553	0.5367	< 0.001
	Within geo-groups	24	78.003	44.72%			
ESGA-ML	Among geo-groups	2	292.330	57.34%	0.573	0.5286	< 0.001
	Within geo-groups	24	281.744	42.66%			

*df* degree of freedom; *SS* square deviation; *PMV* percentages of molecular variance; *F*_*st*_ coefficient of genetic differentiation; *N*_*m*_ gene flow

## Discussion

### Characteristics of *E. sibiricus* draft genome

The development of next-generation sequencing (NGS) provided researchers with an attainable and cheaper method to access the plant genomes, especially for the non-model grass species like *Elymus sibiricus*. Based on Illumina sequencing with fourteen 270 bp insertion size libraries, surveyed draft genome of *E. sibiricus* was de novo sequenced and assembled in this study. The moderate GC content (45.68%) of the draft genome indicated that the Illumina platform was excellently avoided sequencing bias. The final assembly had a N50 of 2510 bp for contigs, far less than that of *Lolium perenne* L. (contig N50 = 16,370 bp) [19], while slightly larger than that of allotetraploid *Arachis hypogaea* L. (contig N50 = 696.6 bp) [20]. This may be caused by the relatively big estimated genome size (6.86 Gb), high repetitiveness (73.23%) of *E. sibiricus* and the short insertion size of library. The estimated genome size of *E. sibiricus* (6.86 Gb) was smaller than that of related allohexaploid *Triticum aestivum* (17 Gb) [21], while larger than that of many other important species in Gramineae, such as *Hordeum vulgare* (5.1 Gb) [22], *Aegilops tauschii* (4.5 Gb) [23], *Triticum urartu* (5.0 Gb) [24], *Brachypodium distachyon* (260 Mb) [25], *Oryza sativa* (466 Mb) [26], *Sorghum bicolor* (730 Mb) [27], *Lolium perenne* (2 Gb) [28] and *Zea mays* (2.3 Gb) [29]. The low level of heterozygosity for *E. sibiricus* (0.01%) obtained via the k-mer analysis was probably caused by the self-pollinating mating system of *E. sibiricus*, and indicated its feasibility for genome sequencing. This is the first draft genome of *E. sibiricus* and it is useful in the molecular marker development and functional gene mining. This work also provided the basis for further whole-genome sequencing using larger insert libraries and new sequencing technique like the single-molecule real-time sequencing.

### SSR marker development

SSR markers have been widely applied in genetic study and molecular breeding. Among all of the identified 293,362 SSRs, the vast of SSRs (97.95%) belonged to mono-, di- and tri-nucleotide motifs, which was similar to the result of restriction site associated DNA-Seq (RAD) in *E. nutans* [30]. However, in the transcriptome sequencing study of *E. sibiricus*, the tri-nucleotide motifs had the largest number [31], which could be due to the difference between sequences in non-coding and coding regions. Typically, the coding regions has a higher percentage of trinucleotides due to the enrichment of triplet codons under selection pressure [32]. Usually, the most abundant tri-nucleotide motif in monocotyledon is CCG/CGG [33], while in this study that is CTC/GAG. This could be the result of codon usage bias in different species [34]. The A/T rich tendency of SSRs in *E. sibiricus* was also consistent with the study of eukaryotes as reported [35]. The phenomenon that motif abundance decreased as the motif repeat number increased of each motif type was in accordance with the previous study [36].

For polyploid species, it’s usually hard to distinguish alleles because of the reciprocal overlapping and uncertain allelism of these fragments [37], which is difficult for genotype scoring. In this case, single-locus SSR markers are considered as the best choice, and development of single-locus SSRs has been reported in barley, peanut and *Luffa* by genome survey [9, 12, 38]. In this study, 10 single-locus SSR markers were developed via the genome survey of *E. sibiricus* with great potential use in genetic variation study and linkage map construction.

### Effectiveness comparison between single- and multi-loci markers

Genetic diversity of 27 wild *E. sibiricus* accessions was evaluated by 30 markers developed in this study and other 30 ones reported before. We found that the expected single-locus SSRs screened by in silico analysis still exhibited multi-loci amplicons when separated by polyacrylamide gel. This may be caused by their non-conservatism of flanking sequences [9]. Finally, only 10 single-locus (ESGA-SL) markers and 20 multi-locus (ESGA-ML) markers were obtained in this study for genetic diversity analysis of 27 wild *E. sibiricus* accessions.

The average amplified alleles of the 10 ESGA-SL markers was 2.9, which was close to the allotetraploid species *Arachis hypogaea* (3.85) and *Brassica napus* (3.23) [9, 37]. The PIC value of the 10 ESGA-SL markers varied from 0.069 to 0.595 with an average of 0.391, that indicated its abundant polymorphism and high application value [39]. There was no significant difference of PIC detected between ESGA-SL and other three marker systems (ESGA-ML, ES and ESGS), which may be caused by the different calculation criteria between single-locus and multi-loci marker or the limited amplification loci of single-locus markers. According to the Mann Whitney test, G-SSR (ESGA and ESGS markers) was more efficient and polymorphic than EST-SSR (ES markers) in view of PIC, MI and Rp, that may be driven by the more conservative flanking sequences of EST-SSR [40, 41]. In addition, significantly (*P* < 0.05) higher PIC values of ESGA-ML markers vs. ESGS markers were calculated, which demonstrate the superiority of SSR markers development method by sequencing over traditional method.

The UPGMA and PCoA derived cluster analysis based on ESGA-SL markers divided the 27 wild *E. sibiricus* accessions into two groups, and the structure analysis based on Bayesian algorithm also revealed the same pattern. However, the other three types of multi-loci markers exhibited inferior ability than ESGA-SL marker in revealing actual genetic relationships. One should note that all the genetic diversity parameters (N_a_, N_e_, I, H_e_ and PP) of each geo-group calculated based on ESGA-SL markers were higher than that of ESGA-ML, which suggested that the single-locus marker reveals more accurate genetic information, so it is more suitable for further genetic analysis [37]. However, slightly higher pairwise F_st_ values were observed among each geo-group based on ESGA-ML markers. Given that multi-loci SSRs possesses characteristic like multiple amplification sites in the genome location, a part of genetic information was unavoidably covered. The advantage of single-locus markers over multi-loci markers was manifested in this study, however, vast number of single-locus markers that covering the entire genome of *E. sibiricus* are required further be identified or developed. In this case, higher quality genome-wide sequencing and assembling for *E. sibiricus* are necessary.

## Conclusions

In this study, the de novo whole genomic survey of *E. sibiricus* was performed and a 4.34 Gb reference genome sequence was obtained with 73.23% repetitive elements, 0.01% heterozygosity and 45.68% GC content. Totally 293,362 SSR markers were identified from the draft genome and 67,833 potential markers were screened by in silico analysis. Subsequently, ten single-locus (ESGA-SL) markers and 20 multi-locus (ESGA-ML) markers were verified by gel electrophoresis and exhibited polymorphism in 27 *E. sibiricus* accessions. The single-locus marker was proved more efficient and informativeness in genetic study than multi-loci marker. This survey of the genome and the developed SSR markers will facilitate further whole genome sequencing, molecular breeding and phylogenetic study of *E. sibiricus* and related Triticeae species.

## Methods

### Plant materials

*E. sibiricus* cultivar ‘Chuancao No.2’ provided by Sichuan Academy of Grassland Sciences (Chengdu, China) was adopted after identification as tetraploid by flow cytometry (Fig. S8) and planted in the growth chamber (25 °C, 300 μmols·m^2^·s^− 1^, 16-h photoperiod). The total genomic DNA of ‘Chuancao No.2’ was isolated from fresh young and clean leaves using a DNA extraction kit (Tiangen, Beijing, China). DNA concentration and purity were checked on a BioPhotometer (Eppendorf, Germany) and the quality was detected by 1% agarose gel electrophoresis.

### Library construction and Illumina sequencing

Fourteen genomic paired-end (PE) libraries with 270-bp insertions were prepared following the manufacturer’s instructions and then sequenced on an Illumina HiSeq X-ten platform. Clean reads were obtained abide by the following filtration and correction criterion [9]: less than 10% unidentified nucleotides (N); no more than 10 nt aligned to the adaptor, allowing for at most 10% mismatches; with at most 50% bases having a phred quality of < 5. Putative PCR duplicates generated during PCR amplification in the library construction process was excluded. In addition, to investigate the potential contaminating effect, 10,000 pairs of clean reads were randomly selected and searched against the NCBI database using BLAST [42]. Finally, to evaluate the content of extra-nuclear DNA in the aforementioned fourteen libraries, BLAST was performed using SOAP [43] with the chloroplast genome of *E. sibiricus* (MK775250, 135,075 bp).

### Genome assembly, annotation and guanine plus cytosine (GC) content analysis

The filtered high-quality sequences were assembled by SOAPdenovo2 [44] following the k-mer size = 54 with default parameters, then GC content was calculated. Identification of protein-coding region and gene prediction of the assembly were conducted through the homology-based prediction method by alignment to genomes of four related species, *Triticum aestivum* [21], *Hordeum vulgare* [45], *Aegilops tauschii* [23] and *T. urartu* [24], and E-value cutoff was set as 1e-5. The GeneWise software [46] was used to predict the exact gene structure of the corresponding genomic regions after removing redundancy. Finally, Trnascan-SE software [46] was applied to predict tRNA.

### Identification and verification of SSRs

Repeat sequence annotation of the newly obtained genome sequence set of *E. sibiricus* was carried out by RepeatMasker [16] following the repeat sequence database of Gramineae [47]. Then PERL5 script microsatellite software (http://pgrc.ipk-gatersleben.de/misa/) was used to identify SSRs in the genomic DNA sequences. The recognition criteria are as follows: the number of single nucleotide repeats is 8 or more; the number of di-, tri-, tetra-, penta- and hexa-nucleotides repeats are all more than 5 [48]. The parameters setting of primer design was: 18 ~ 27 bp primer size, annealing temperature at 55–65 °C, GC content at 30–70% and 100 ~ 300 bp final product length [17]. Using in silico analysis, the designed primers were mapped back onto the assembly sequence of ‘Chuancao NO.2’, and the SSR combined with only one site was regarded as potential single-locus SSR [49].

### SSRs evaluation based on the genetic diversity of *E. sibiricus* germplasm

Four hundred pairs of SSR markers were randomly selected for synthesis, then PCR and electrophoresis were performed for screening and validation. Primers with only 0–2 amplified bands were recognized as single-locus SSR and those possessing polymorphism was named ‘*E. sibiricus* genome assembly single locus’ (ESGA-SL) marker. Analogously, primers with more than 2 amplified bands simultaneously polymorphic was called ‘*E. sibiricus* genome assembly multi loci’ (ESGA-ML) marker. In addition, 30 pairs of multi loci markers including fifteen G-SSR markers (ESGS [40]) developed based on magnetic bead enrichment, and fifteen pairs of EST-SSR markers (ES [31]) based on *E. sibiricus* transcriptome were also selected to amplified the same 27 *E. sibiricus* accessions DNA (Table S5, Supplementary file).

Amplified bands were recorded following genotypes (single locus marker) or 0/1 binary matrix (multi loci marker). Nei’s genetic distance (GD) matrix among 27 accessions was calculated by Freetree [50] with 10,000 bootstrap value, and the UPGMA dendrogram was constructed then visualized in Figtree [50]. The principal coordinate analysis (PCoA) was performed via NTSYS v2.2 [51]. STRUCTURE v2.3.4 [52] was performed based on a Bayesian model to the illustration of genetic membership. The parameters were set to 50,000 burn-in and 100,000 Monte Carlo Markov chain (MCMC) with an admixture model. The STRUCTURE HARVESTER [53] was then applied to estimate the “optimum K”. The hierarchical analysis of molecular variance (AMOVA) was carried out using GenAlEx [54] to calculate the number of alleles (N_a_), effective alleles (N_e_), Shannon diversity index (I), expected heterozygosity (H_e_), polymorphic site proportion (PP) and other genetic diversity parameters. Finally, the observed heterozygosity (H_o_) and gene flow (N_m_) of single-locus SSRs were calculated.

## Supplementary Information

**Additional file 1: Table S1.** Predicted gene information of *E. sibiricus* and its related species.

**Additional file 2: Table S2.** Length and ratio of repetitive elements in *E. sibiricus*.

**Additional file 3: Table S3.** Statistics of SSRs in *E. sibiricus* genome.

**Additional file 4: Table S4.** Statistics of SSR motif with different repeat number.

**Additional file 5: Table S5.** Wild *E. sibiricus* accessions used in this study.

**Additional file 6: Table S6.** SSR primers used in this study.

**Additional file 7: Table S7.**. Genetic indexes of multi loci markers.

**Additional file 8: Table S8.** Statistical significance of Mann-Whitney test among PIC, MI, BI and Rp of different markers

**Additional file 9: Figure S1.** Example of PAGE electrophoretic picture of single-loci (ESGA-SL-2 and ESGA-SL-9) and multi-locus (ESGA-ML-7 and ESGA-ML-10) markers in this study.

**Additional file 10: Figure S2**. The UPGMA dendrogram and genetic structure of 27 studied *E. sibiricus* based on ESGA-ML markers.

**Additional file 11: Figure S3**. The PcoA analysis of 27 studied *E. sibiricus* based on ESGA-ML markers.

**Additional file 12: Figure S4.** The UPGMA dendrogram and genetic structure of 27 studied *E. sibiricus* based on ESGS markers.

**Additional file 13: Figure S5.** The PcoA analysis of 27 studied *E. sibiricus* based on ESGS markers.

**Additional file 14: Figure S6.** The UPGMA dendrogram and genetic structure of 27 studied *E. sibiricus* based on ES markers.

**Additional file 15: Figure S7.** The PcoA analysis of 27 studied *E. sibiricus* based on ES markers.

**Additional file 16: Figure S8.** *E. sibiricus* cultivar ‘Chuancao No.2’ was identified as tetraploid by flow cytometry. Red color is diploid *Hordeum vulgare* L., yellow is *E. sibiricus* cultivar ‘Chuancao No.2’ and pink is hexaploid *Triticum aestivum*.

## Data Availability

All datasets supporting the conclusions of this article are included within the article and its additional file. The raw DNA sequencing data are available on the NCBI SRA database with the citation accession ‘PRJNA680324’.

## Abbreviations

NGS: Next-generation sequencing
QTP: Qinghai-Tibet Plateau
FCM: Flow cytometry
SSRs: Simple sequence repeats
ESTs: Expressed sequence tags
G-SSR: Genomic DNA-derived SSRs
ESGA-SL: *E. sibiricus* genome assembly single locus marker
ESGA-ML: *E. sibiricus* genome assembly multi loci marker

## References

[1] Ma X, Chen SY, Zhang XQ, Zhou YH, Bai SQ, Liu W (2009). Genetic diversity of gliadin in worldwide germplasm collections of *Elymus sibiricus*. Acta Pratacul Sin.

[2] Dewey DR, Barkworth ME (1984). The genomic system of classification as a guide to intergeneric hybridization with the perennial Triticeae. Genet. Manipulation Plant Breed., Proc., Int. Symp.

[3] Yan JJ, Bai SQ, Ma X, Gan YM, Zhang JB (2007). Genetic diversity of *Elymus sibiricus* and its breeding in China. Chin Bull Bot.

[4] Ma X, Zhang XQ, Zhou YH, Bai SQ, Liu W (2008). Assessing genetic diversity of *Elymus sibiricus* (Poaceae: Triticeae) populations from Qinghai-Tibet plateau by ISSR markers. Biochem Syst Ecol.

[5] Yan JJ, Bai SQ, Zhang XQ, You MH, Zhang CB, Li DX, Zeng Y (2010). Genetic diversity of wild *Elymus sibiricus* germplasm from the Qinghai-Tibetan plateau in China detected by SRAP markers. Acta Pratacul Sin.

[6] Vogel KP, Arumuganathan K, Jensen KB (1999). Nuclear DNA content of perennial grasses of the Triticeae. Crop Sci.

[7] Zhou W, Hu YY, Sui ZH, Fu F, Wang JG, Chang LP, Guo WH, Li BB (2013). Genome survey sequencing and genetic background characterization of *Gracilariopsis lemaneiformis* (Rhodophyta) based on next-generation sequencing. PLoS One.

[8] Jiang GL (2015). Molecular marker-assisted breeding: a plant breeder's review.

[9] Zhou XJ, Dong Y, Zhao JJ, Huang L, Ren XP, Chen YN, Huang SM, Liao BS, Lei Y, Yan LY, Jiang HF (2016). Genomic survey sequencing for development and validation of single-locus SSR markers in peanut (*Arachis hypogaea* L.). BMC Genomics.

[10] Sharma MK, Sharma R, Cao PJ, Jenkins J, Bartley LE, Qualls M, Grimwood J, Schmutz J, Rokhsar D, Ronald PC (2012). A genome-wide survey of switchgrass genome structure and organization. PLoS One.

[11] Xiao J, Zhao J, Liu MJ, Liu P, Dai L, Zhao ZH (2015). Genome-wide characterization of simple sequence repeat (SSR) loci in chinese jujube and jujube SSR primer transferability. PLoS One.

[12] An JY, Yin MQ, Zhang Q, Gong DT, Jia XW, Guan YJ, Hu J (2017). Genome survey sequencing of *Luffa Cylindrica* L. and microsatellite high resolution melting (SSR-HRM) analysis for genetic relationship of *Luffa* genotypes. Int J Mol Sci.

[13] Fluch S, Burg A, Kopecky D, Homolka A, Spiess N, Vendramin GG (2011). Characterization of variable EST SSR markers for Norway spruce (*Picea abies* L.). BMC Res. Notes.

[14] Zhang Q, Li J, Zhao YB, Korban SS, Han YP (2012). Evaluation of genetic diversity in Chinese wild apple species along with apple cultivars using SSR markers. Plant Mol Biol Rep.

[15] Chen S, Nelson MN, Ghamkhar K, Fu T, Cowling WA (2008). Divergent patterns of allelic diversity from similar origins: the case of oilseed rape (*Brassica napus* L.) in China and Australia. Genome.

[16] Tempel S (2012). Using and understanding RepeatMasker.

[17] Untergasser A, Cutcutache I, Koressaar T, Ye J, Faircloth BC, Remm M, Rozen SG (2012). Primer3--new capabilities and interfaces. Nucleic Acids Res.

[18] Rahardja D, Zhao YD, Qu Y (2009). Sample size determinations for the wilcoxon–mann–Whitney test: a comprehensive review. Stats in Biopharmaceutical Research.

[19] Byrne SL, Nagy I, Pfeifer M, Armstead I, Swain S, Studer B, Mayer K, Campbell JD, Czaban A, Hentrup S, Panitz F, Bendixen C, Hedegaard J, Caccamo M, Asp T (2015). A synteny-based draft genome sequence of the forage grass *Lolium perenne*: for cell and molecular biology. Plant J.

[20] Bertioli DJ, Jenkins J, Clevenger J, Dudchenko O, Gao DC, Seijo G, Leal-Bertioli SCM, Ren LH, Farmer AD, Pandey MK (2019). The genome sequence of segmental allotetraploid peanut *Arachis hypogaea*. Nat Genet.

[21] Zimin AV, Puiu D, Hall R, Kingan S, Clavijo BJ, Salzberg SL. The first near-complete assembly of the hexaploid bread wheat genome. *Triticum aestivum* Gigascience. 2017;6:1–7. 10.1093/gigascience/gix097 PMC5691383 29069494

[22] Mascher M, Gundlach H, Himmelbach A, Beier S, Twardziok SO, Wicker T, Radchuk V, Dockter C, Hedley PE, Russell J (2017). A chromosome conformation capture ordered sequence of the barley genome. Nature..

[23] Zhao GY, Zou C, Li K, Wang K, Li TB, Gao LF, Zhang XX, Wang HJ, Yang ZJ, Liu X (2017). The *Aegilops tauschii* genome reveals multiple impacts of transposons. Nat Plants.

[24] Ling HQ, Zhao SC, Liu DC, Wang JY, Sun H, Zhang C, Fan HJ, Li D, Dong LL, Tao Y (2013). Draft genome of the wheat A-genome progenitor *Triticum urartu*. Nature.

[25] The International Brachypodium Initiative (2010). Genome sequencing and analysis of the model grass *Brachypodium distachyon*. Nature..

[26] International Rice Genome Sequencing Project (2005). The map-based sequence of the rice genome. Nature..

[27] Paterson AH, Bowers JE, Bruggmann R, Dubchak I, Grimwood J, Gundlach H, Haberer G, Hellsten U, Mitros T, Poliakov A (2009). The *Sorghum bicolor* genome and the diversification of grasses. Nature..

[28] Byrne S, Panitz F, Hedegaard J, Bendixen C, Studer B, Farrell JD, Swain S, Armstead I, Caccamo M, Asp T. De novo genome sequencing of perennial ryegrass (*Lolium perenne*). Int Plant & Animal Genome Conference XX. 2011.

[29] Jiao YP, Peluso P, Shi JH, Liang T, Stitzer MC, Wang B, Campbell MS, Stein JC, Wei XH, Chin CS (2017). Improved maize reference genome with single-molecule technologies. Nature..

[30] Liu RJ, Lu XW, Dou QW (2018). Development of SSR markers in *Elymus nutans* based on reduced-representation genome sequencing. Mol Plant Breed.

[31] Zhou Q, Luo D, Ma LC, Xie WG, Wang Y, Wang YR, Liu ZP (2016). Development and cross-species transferability of EST-SSR markers in Siberian wildrye (*Elymus sibiricus* L.) using Illumina sequencing. Sci. Rep.

[32] Blanca J, Canizares J, Roig C, Ziarsolo P, Nuez F, Picó B (2011). Transcriptome characterization and high throughput SSRs and SNPs discovery in *Cucurbita pepo* (Cucurbitaceae). BMC Genomics.

[33] Kantety RV, Rota ML, Matthews DE, Sorrells ME (2002). Data mining for simple sequence repeats in expressed sequence tags from barley, maize, rice, sorghum and wheat. Plant Mol Biol.

[34] Qin Z, Cai ZQ, Xia GM, Wang MC (2013). Synonymous codon usage bias is correlative to intron number and shows disequilibrium among exons in plants. BMC Genomics.

[35] Tóth G, Gáspári Z, Jurka J (2000). Microsatellites in different eukaryotic genomes: survey and analysis. Genome Res.

[36] Shulaev V (2011). The genome of woodland strawberry (*Fragaria vesca*). Nat Genet.

[37] Li HT, Younas M, Wang XF, Li XM, Chen L, Zhao B, Chen X, Xu JS, Hou F, Hong BH (2013). Development of a core set of single-locus SSR markers for allotetraploid rapeseed (*Brassica napus* L.). Theor. Appl. Genet..

[38] Varshney RK, Thiel T, Sretenovic-Rajicic T, Baum M, Valkoun J, Guo P, Grando S, Ceccarelli S, Graner A (2008). Identification and validation of a core set of informative genic SSR and SNP markers for assaying functional diversity in barley. Mol Breed.

[39] Botstein D, White RL, Skolnick M (1980). Construction of a genetic linkage map in man using restriction fragment length polymorphisms. Am J Hum Genet.

[40] Lei YT, Zhao YY, Yu F, Li Y, Dou QW (2014). Development and characterization of 53 polymorphic genomic-SSR markers in Siberian wildrye (*Elymus sibiricus* L.). Conserv Genet Resour.

[41] Eujayl I, Sorrells ME, Baum M, Wolters P, Powell W (2002). Isolation of EST-derived microsatellite markers for genotyping the a and B genomes of wheat. Theor Appl Genet.

[42] Altschul SF (1990). Basic local alignment search tool (BLAST). J Mol Biol.

[43] Li RQ, Li YR, Kristiansen K, Wang J (2008). SOAP: short oligonucleotide alignment program. Bioinformatics..

[44] Luo RB, Liu BH, Xie YL, Li ZY, Huang WH, Yuan JY, He GZ, Chen YX, Pan Q, Liu YJ (2012). SOAPdenovo2: an empirically improved memory-efficient short-read de novo assembler. Gigascience..

[45] The International Barley Genome Sequencing Consortium (2012). A physical, genetic and functional sequence assembly of the barley genome. Nature..

[46] Lowe TM, Chan PP (2016). tRNAscan-SE on-line: integrating search and context for analysis of transfer RNA genes. Nucleic Acids Res.

[47] Bao W, Kojima KK, Kohany O (2015). Repbase update, a database of repetitive elements in eukaryotic genomes. Mob DNA.

[48] Thiel T, Michalek W, Varshney R, Graner A (2003). Exploiting EST databases for the development and characterization of gene-derived SSR-markers in barley (*Hordeum vulgare* L.). Theor. Appl. Genet.

[49] Schuler GD (1997). Sequence mapping by electronic PCR. Genome Res.

[50] Hampl V, Pavlicek A, Flegr J (2001). Construction and bootstrap analysis of DNA fingerprinting-based phylogenetic trees with the freeware program FreeTree: application to trichomonad parasites. Int J Syst Evol Microbiol.

[51] Rohlf FJ (1987). NTSYS-pc: microcomputer programs for numerical taxonomy and multivariate analysis. Am Stat.

[52] Falush D, Stephens M, Pritchard JK (2007). Inference of population structure using multilocus genotype data: dominant markers and null alleles. Mol Ecol Notes.

[53] Earl DA, Vonholdt BM (2012). STRUCTURE HARVESTER: a website and program for visualizing STRUCTURE output and implementing the Evanno method. Conserv Genet Resour.

[54] Peakall R, Smouse PE (2006). Genalex 6: genetic analysis in excel. Population genetic software for teaching and research. Mol. Ecol. Notes..

