# Supplementary material for: Genomic survey sequencing, development and characterization of single- and multi-locus genomic SSR markers of Elymus sibiricus L
Source: BMC Plant Biol. 2021 Jan 6;21:3. doi: 10.1186/s12870-020-02770-0 (PMC7789342; doi:10.1186/s12870-020-02770-0)
Supplement: Supplementary file 1 — Additional file 1: Table S1. Predicted gene information of E. sibiricus and its related species. [file 12870_2020_2770_MOESM1_ESM.docx]

**Table S1** Predicted gene information of *E. sibiricus* and its related species.

| Species | Gene number | Average transcript length (bp) | Average CDS length (bp) | Average exons per gene | Average exon length (bp) | Average intron length (bp) |
| --- | --- | --- | --- | --- | --- | --- |
| *Elymus sibiricus* | 25,993 | 2,632.11 | 737.36 | 4.72 | 311.32 | 347.67 |
| *Triticum aestivum* | 58,175 | 2,267.84 | 1,013.79 | 4.43 | 228.96 | 365.84 |
| *Hordeum vulgare* | 21,644 | 2,285.16 | 1,140.94 | 4.39 | 259.69 | 337.18 |
| *Aegilops tauschii* | 35,138 | 2,230.08 | 1,119.96 | 4.41 | 253.77 | 325.18 |
| *Triticum Urartu* | 33,442 | 3,268.84 | 1,090.78 | 4.77 | 228.38 | 576.76 |
