# Supplementary material for: Genomic survey sequencing, development and characterization of single- and multi-locus genomic SSR markers of Elymus sibiricus L
Source: BMC Plant Biol. 2021 Jan 6;21:3. doi: 10.1186/s12870-020-02770-0 (PMC7789342; doi:10.1186/s12870-020-02770-0)
Supplement: Supplementary file 2 — Additional file 2: Table S2. Length and ratio of repetitive elements in E. sibiricus. [file 12870_2020_2770_MOESM2_ESM.docx]

**Table S2** Length and ratio of repetitive elements in *E. sibiricus.*

| Repeat | Length occupied (bp) | Percentage (%) | |
| --- | --- | --- | --- |
| **Retroelements** | 137,216,460 | 16.45 |  |
| SINEs | 333,657 | 0.04 |  |
| LINEs | 11,427,754 | 1.37 |  |
| RTE/Bov-B | 333,657 | 0.04 |  |
| L1/CIN4 | 12,678,968 | 1.52 |  |
| LTR elements | 132,628,675 | 15.90 |  |
| Ty1/Copia | 34,366,676 | 4.12 |  |
| Gypsy/DIRS1 | 96,176,643 | 11.53 |  |
| **DNA transposons** | 27,193,049 | 3.26 |  |
| hobo-Activator | 1,167,800 | 0.14 |  |
| Tc1-IS630-Pogo | 2,085,357 | 0.25 |  |
| Tourist/Harbinger | 1,751,699 | 0.21 |  |
| **Unclassified** | 917,557 | 0.11 |  |
| **Small RNA** | 917,557 | 0.11 |  |
| **Satellites** | 417,071 | 0.05 |  |
| **Simple repeats** | 7,006,798 | 0.84 |  |
| **Low complexity** | 1,501,457 | 0.18 |  |
