# Supplementary material for: Genomic survey sequencing, development and characterization of single- and multi-locus genomic SSR markers of Elymus sibiricus L
Source: BMC Plant Biol. 2021 Jan 6;21:3. doi: 10.1186/s12870-020-02770-0 (PMC7789342; doi:10.1186/s12870-020-02770-0)
Supplement: Supplementary file 3 — Additional file 3: Table S3. Statistics of SSRs in E. sibiricus genome. [file 12870_2020_2770_MOESM3_ESM.docx]

**Table S3** Statistics of SSRs in *E. sibiricus* genome.

|  | Number | Ratio |
| --- | --- | --- |
| Total number of examined sequences | 2,506,979 (4,337,715,088 bp) | 100.00% |
| Number of SSR containing sequences | 507,162 | 20.23% |
| Number of sequences containing single-locus SSR | 22,611 | 0.90% |
| Total number of SSRs | 315,446 | 100.00% |
| Number of SSRs present in compound form | 22,084 | 7.00% |
