# Supplementary material for: Genomic survey sequencing, development and characterization of single- and multi-locus genomic SSR markers of Elymus sibiricus L
Source: BMC Plant Biol. 2021 Jan 6;21:3. doi: 10.1186/s12870-020-02770-0 (PMC7789342; doi:10.1186/s12870-020-02770-0)
Supplement: Supplementary file 4 — Additional file 4: Table S4. Statistics of SSR motif with different repeat number. [file 12870_2020_2770_MOESM4_ESM.docx]

**Table S4** Statistics of SSR motif with different repeat number.

| SSR motif repeat number | Total number of SSR motifs |
| --- | --- |
| 5 | 30523 |
| 6 | 31980 |
| 7 | 14921 |
| 8 | 8835 |
| 9 | 5406 |
| 10 | 77930 |
| 11 | 31575 |
| 12 | 18664 |
| 13 | 11763 |
| 14 | 8226 |
| 15 | 6312 |
| 16 | 4925 |
| 17 | 3818 |
| 18 | 2882 |
| 19 | 2638 |
| 20 | 2485 |
| ＞20 | 30479 |
