# Supplementary material for: Genomic survey sequencing, development and characterization of single- and multi-locus genomic SSR markers of Elymus sibiricus L
Source: BMC Plant Biol. 2021 Jan 6;21:3. doi: 10.1186/s12870-020-02770-0 (PMC7789342; doi:10.1186/s12870-020-02770-0)
Supplement: Supplementary file 5 — Additional file 5: Table S5. Wild E. sibiricus accessions used in this study. [file 12870_2020_2770_MOESM5_ESM.docx]

**Table S5** Wild accessions of *E. sibiricus* used in this study.

|  | **Accession ID** | **Country** | **Geographical region** | **Source** | |
| --- | --- | --- | --- | --- | --- |
| 01 | **PI 610866** | Mongolia | Mongolia | NPGS | |
| 02 | **PI 610876** | Mongolia | Mongolia | NPGS |  |
| 03 | PI 610850 | Mongolia | Mongolia | NPGS | |
| 04 | PI 634231 | Mongolia | Mongolia | NPGS | |
| 05 | PI 610857 | Mongolia | Mongolia | NPGS | |
| 06 | PI 610862 | Mongolia | Mongolia | NPGS | |
| 07 | PI 610860 | Mongolia | Mongolia | NPGS | |
| 08 | PI 610886 | Mongolia | Mongolia | NPGS | |
| 09 | **SAU07001** | China | Eastern Qinghai-Tibet Plateau | Artificial acquisition | |
| 10 | SAU07011 | China | Eastern Qinghai-Tibet Plateau | Artificial acquisition | |
| 11 | **SAU07013** | China | Eastern Qinghai-Tibet Plateau | Artificial acquisition | |
| 12 | **SAU07008** | China | Eastern Qinghai-Tibet Plateau | Artificial acquisition | |
| 13 | SAU07025 | China | Eastern Qinghai-Tibet Plateau | Artificial acquisition | |
| 14 | **PI 499456** | China | Eastern Qinghai-Tibet Plateau | NPGS | |
| 15 | SAU07022 | China | Eastern Qinghai-Tibet Plateau | Artificial acquisition | |
| 16 | SAU07007 | China | Eastern Qinghai-Tibet Plateau | Artificial acquisition | |
| 17 | SAU07030 | China | Eastern Qinghai-Tibet Plateau | Artificial acquisition | |
| 18 | SAU07028 | China | Eastern Qinghai-Tibet Plateau | Artificial acquisition | |
| 19 | SAU07017 | China | Eastern Qinghai-Tibet Plateau | Artificial acquisition | |
| 20 | SAU07004 | China | Eastern Qinghai-Tibet Plateau | Artificial acquisition | |
| 21 | SAU07015 | China | Eastern Qinghai-Tibet Plateau | Artificial acquisition | |
| 22 | **PI 598799** | Russia | Siberia | NPGS | |
| 23 | **PI 326267** | Russia | Siberia | NPGS | |
| 24 | PI 611020 | Russia | Siberia | NPGS | |
| 25 | PI 598774 | Russia | Siberia | NPGS | |
| 26 | PI 325315 | Russia | Siberia | NPGS | |
| 27 | PI 598786 | Russia | Siberia | NPGS | |

Note: Code names of eight accessions used to test the polymorphism of primers are in bold. NPGS is the abbreviation for National Plant Germplasm System of USDA.
