# Supplementary material for: Genomic survey sequencing, development and characterization of single- and multi-locus genomic SSR markers of Elymus sibiricus L
Source: BMC Plant Biol. 2021 Jan 6;21:3. doi: 10.1186/s12870-020-02770-0 (PMC7789342; doi:10.1186/s12870-020-02770-0)
Supplement: Supplementary file 6 — Additional file 6: Table S6. SSR primers used in this study. [file 12870_2020_2770_MOESM6_ESM.docx]

**Table S6** SSR primers used in this study.

| **Primer ID** | **Type** | **Source** | **Repeat motif** | **Forward** | **Reverse** | **Tm (℃)** |
| --- | --- | --- | --- | --- | --- | --- |
| ESGA-SL-1 | Single-locus | Genomic | (TTG)_5_ | CATCTGCAATATGCGTTGCT | CAACCAAAAGCACCACAAGA | 58.0 |
| ESGA-SL-2 | Single-locus | Genomic | (CGA)_6_ | GAGGACAGGGTGCAGTGATT | CTGACGTTGGAATGGAGACA | 58.0 |
| ESGA-SL-3 | Single-locus | Genomic | (ATGG)_5_ | GTTGCAGAATGCATTTCCCT | GCGGCGAAAATTATGTCTGT | 58.9 |
| ESGA-SL-4 | Single-locus | Genomic | (AG)_10_c(GA)_11_ | GGTGGAGAAGGGAGATGAGTC | AGGCTCATGAGGAACAAGTCTCT | 58.9 |
| ESGA-SL-5 | Single-locus | Genomic | (AAT)_6_(AATA)_6_ | CTGCCACTTTTGACCTGTCTC | GCCTTTCTGTCTCCTGTACCATA | 56.1 |
| ESGA-SL-6 | Single-locus | Genomic | (AC)_25_ | GAATGGAGCTCCGCTTTAAGATTT | GCTCCAAACACGGTATAACTCCAC | 58.8 |
| ESGA-SL-7 | Single-locus | Genomic | (GCG)_5_ | TTATGTGATTTCATAATTGGCCC | GCTGCTGCTACCGTTCTTATTTA | 59.9 |
| ESGA-SL-8 | Single-locus | Genomic | (AG)_25_angagaggggactgctct(AG)_17_ | GGGTGTGATTCATAAAACGAATG | TCTTTCTCGTGACTGTTCCTTTC | 59.5 |
| ESGA-SL-9 | Single-locus | Genomic | (TG)_8_(G)_27_ | CGTCTTCCGCTTCATCTTCTT | CAAAGATCCAGATCACACCAAAC | 59.5 |
| ESGA-SL-10 | Single-locus | Genomic | (T)_10_accccgc(A)_13_ | GAGGGCATCCAGTACGACTC | CAGGAGCGGGACTAACTGAA | 59.5 |
| ESGA-1 | Multi-loci | Genomic | (TTG)_7_ | CCCCAACTTGTTTGGGACTA | CAGGCGGGATATAGCCAGTA | 52.8 |
| ESGA-2 | Multi-loci | Genomic | (TCA)_5_ | GCACAATGTGTAGCACATAACTCA | CCAGCAACAGCAGACCTACA | 54.9 |
| ESGA-3 | Multi-loci | Genomic | (GTC)_5_ | GCCGGTGAGAGTTCACAAGT | CGGTCGATCGAGATTCACTT | 54.3 |
| ESGA-4 | Multi-loci | Genomic | (TTC)_12_ | GGGGGACAGTCAAAGAAACA | CAACAGTGCCCCTTTGAAGT | 54.8 |
| ESGA-5 | Multi-loci | Genomic | (GGA)_5_ | GTAGGGCGTGAAATGCTTGT | CGATCACCAACACTAAGCGA | 53.2 |
| ESGA-6 | Multi-loci | Genomic | (GCG)_6_ | GTGGGAGAAGACGACCATGT | CCATTTGTCGACCCTGTTTT | 53.1 |
| ESGA-7 | Multi-loci | Genomic | (ATG)_5_ | CTGTTTTCCAGCCTAGGGGT | GAATCACCCACCAAAGGAGA | 54.5 |
| ESGA-8 | Multi-loci | Genomic | (CGT)_5_ | CGTGGGGCTGGAGTAGAATA | GGGGTAGACCGAACTTGACA | 53.9 |
| ESGA-9 | Multi-loci | Genomic | (CAA)_5_ | CGACCTACATCAGGTGCCTT | GCAGCCGTCTCCAGGTACTA | 54.8 |
| ESGA-10 | Multi-loci | Genomic | (TGT)_8_ | CCCAGATCCTCTGGACTTTG | CATAGGGGAGCACTGGAAAA | 56.9 |
| ESGA-11 | Multi-loci | Genomic | (TCG)_5_ | GAAAGTCCACGGGAGAGTCA | CGGAGCTTAGCTACTTGCGT | 55.0 |
| ESGA-12 | Multi-loci | Genomic | (CGG)_6_ | GGCTGAGGGCGGAGTACA | GCAGTACACCCAGTTCAGCA | 56.4 |
| ESGA-13 | Multi-loci | Genomic | (TAA)_6_ | GACATTGTTTACGGCGGTTT | CCTCGGGCATCTCAAGAATA | 56.5 |
| ESGA-14 | Multi-loci | Genomic | (AAC)_6_ | GGCTGGCTAGTGTGATGGAT | CCCCAACTTGTTTGGGACTA | 54.2 |
| ESGA-15 | Multi-loci | Genomic | (TCC)_8_ | GCTCGAAATGCTTGTGTTCA | GGTGGCATTGGTGGGTAATA | 57.2 |
| ESGA-16 | Multi-loci | Genomic | (GTT)_5_ | GTGATTTGGGAGCTGGTGTT | GATGAGGACGGCCGTAAATA | 54.5 |
| ESGA-17 | Multi-loci | Genomic | (AAC)_6_ | GAGGTGTGCCTCCCATTTTA | CAAGCACTCCATCAATGCAA | 55.0 |
| ESGA-18 | Multi-loci | Genomic | (ATA)_6_ | CTACAGGGTCCGTGTTCGAT | GTCATCGTCGTCCTCCTCAT | 57.1 |
| ESGA-19 | Multi-loci | Genomic | (AGA)_5_ | CTTATTGCCGACGTGAGGTT | GGAGGTAGAGGTCGCGGT | 57.3 |
| ESGA-20 | Multi-loci | Genomic | (CTT)_10_ | GCTTTGTTTCCTCCACAATGA | CTCCCCTCCCACTCCATAAT | 56.8 |
| ES-4 | Multi-loci | Transcriptomic | (CGC)_5_ | GGTAGACCCCCACGATCTC | AGTTGACTAGTGCTCATTCGCTC | 60.0 |
| ES-5 | Multi-loci | Transcriptomic | (GAT)_5_ | CGTCTCCAAGGTGATGAAGTTT | ATCAGTGAAATGTGTCACACCC | 60.0 |
| ES-8 | Multi-loci | Transcriptomic | (GCT)_5_ | GTTCATCCTCCCCTTCAGATTC | GTACAAATCCCAGACCTGAGAAC | 60.0 |
| ES-23 | Multi-loci | Transcriptomic | (CCG)_5_ | CGTACTTGCGCCAGAAGTG | AGGTGTCCATCGAAGGGTC | 60.0 |
| ES-24 | Multi-loci | Transcriptomic | (TG)_8_ | AGTTGCTAGTTGTGCTTGTGTCA | CATCTGCGTACAAAACTGTGAAA | 60.0 |
| ES-25 | Multi-loci | Transcriptomic | (TGC)_6_ | AGCGATGATGTGAACAAGTAGGT | CTGACAAATACAGATGCACCAGA | 60.0 |
| ES-40 | Multi-loci | Transcriptomic | (GGC)_6_ | TACTACCGAGGTATGTACAGGGG | GAAGAGGGCGAAGAAGGC | 60.0 |
| ES-41 | Multi-loci | Transcriptomic | (GGC)_5_ | GTAAAACGTTGCCTGGTCCTC | CTCCAGCTGCAGAAACAGC | 60.0 |
| ES-86 | Multi-loci | Transcriptomic | (GCC)_5_ | CATTGTTACATTGCACAGCAGAT | CATGTTGAATGGTCCTCTTGG | 60.0 |
| ES-91 | Multi-loci | Transcriptomic | (CGC)_5_ | AGTCGGTGACATACAAGCAATG | TTAATACCAGTAGCTTCCGCTTC | 60.0 |
| ES-103 | Multi-loci | Transcriptomic | (GCG)_5_ | GTTAGAGAAGGAGGCGGTATGG | CACTTCTCACTGTCCACTCCAAG | 61.0 |
| ES-105 | Multi-loci | Transcriptomic | (GGA)_5_ | GGTGGAGAAGGGAGATGAGTC | AGGCTCATGAGGAACAAGTCTCT | 60.0 |
| ES-123 | Multi-loci | Transcriptomic | (GAA)_5_ | AGCATGAAGCTCGACTGTGAGT | GCGAGTACATCTCGTACTTCTGG | 61.0 |
| ES-141 | Multi-loci | Transcriptomic | (TTC)_6_ | TTATGTGATTTCATAATTGGCCC | GCTGCTGCTACCGTTCTTATTTA | 60.0 |
| ES-149 | Multi-loci | Transcriptomic | (AGA)_5_ | AGGAATTCAACCAAGAGGAGC | AAGAGCATGCTGGTGAGGATAC | 60.0 |
| ESGS-1 | Multi-loci | Genomic | (CT)_36_ | GGTGCTGTTTGTTTGTCT | GAATGAAAGTTGCGGGTT | 49.6 |
| ESGS-4 | Multi-loci | Genomic | (GA)_14_ | GTGCCCTATCAAGATTACG | TTCATCGGGACACCTTTT | 53.0 |
| ESGS-6 | Multi-loci | Genomic | (GA)_12_–(GA)_14_ | ACCTAAGCAAAGCCAGAT | CAGTGATTGATGAGTCGC | 48.8 |
| ESGS-31 | Multi-loci | Genomic | (TATG)_4_ | GGAGATCACAGACACAATAATTCTA | TGATTCGTATTTTTCTTATTAGTCT | 50.5 |
| ESGS-41 | Multi-loci | Genomic | (CT)_14_ | TACATCCACAACTTGAGCACC | CACAACTCACAAGCAGGACAC | 52.3 |
| ESGS-55 | Multi-loci | Genomic | (CT)_12_ | CTGAATTATTTTGACATT | TGTAACATTTGAGAGAGA | 43.6 |
| ESGS-87 | Multi-loci | Genomic | (GA)_13_ | AAGTGCCAACTAGGAGTTTG | ACATCACCATTTTACAGGGA | 47.2 |
| ESGS-90 | Multi-loci | Genomic | (TC)_11_–(CT)_6_ | ATCCCTCCACCTAAATCTGTACC | AAGAATCTAAAAATCATCGACGA | 50.7 |
| ESGS-98 | Multi-loci | Genomic | (CT)_7_ | AAAGAAAAGGAAATAAACG | GGAGGTGGGAGACGAACTG | 50.9 |
| ESGS-117 | Multi-loci | Genomic | (CT)_9_ | CGAGGCGAGGTAAAAAGTATA | GTTGTCACGTTTGAAGCAAGT | 53.9 |
| ESGS-142 | Multi-loci | Genomic | (GA)_16_ | AAATAAGAGGTAGGGAGGCT | TGATTGTAGGGGAAAACAAG | 51.7 |
| ESGS-170 | Multi-loci | Genomic | (AC)_7_–(CAC)_5_ | CATGAGAGGCATAGCTCCCAC | AATTTACAAACGCTCGCGTTC | 55.7 |
| ESGS-172 | Multi-loci | Genomic | (GA)_7_ | TTGAAGCAAGTACAACTA | GTAAAATCTACGGAAAGC | 49.8 |
| ESGS-183 | Multi-loci | Genomic | (GA)_7_ | GTCACGTTTGAAGCAAGTA | GGCGAGGTAAAAAGTATAG | 52.0 |
| ESGS-193 | Multi-loci | Genomic | (AC)_10_ | CAAAATAAATTGGTGCGTTG | CTGCTTCCTCCCTTTCTACA | 48.9 |
