# Supplementary material for: Genomic survey sequencing, development and characterization of single- and multi-locus genomic SSR markers of Elymus sibiricus L
Source: BMC Plant Biol. 2021 Jan 6;21:3. doi: 10.1186/s12870-020-02770-0 (PMC7789342; doi:10.1186/s12870-020-02770-0)
Supplement: Supplementary file 7 — Additional file 7: Table S7.. Genetic indexes of multi loci markers. [file 12870_2020_2770_MOESM7_ESM.docx]

**Table S7** Genetic indexes of multi loci markers.

| Type | Statistics | TNB | NPB | PPB | PIC | MI | BI | Rp |
| --- | --- | --- | --- | --- | --- | --- | --- | --- |
| ESGA-ML | Total | 115 | 105 | / | / | 42.2611 | 12.9882 | 67.7779 |
|  | Minimum | 4 | 2 | 50.00% | 0.3468 | 0.9164 | 0.5103 | 1.5556 |
|  | Minimum Primer | ESGA-ML-1/10/18 | ESGA-ML-10 | ESGA-ML-10 | ESGA-ML-5 | ESGA-ML-10 | ESGA-ML-3 | ESGA-ML-10 |
|  | Maximum | 9 | 9 | 100.00% | 0.4664 | 3.2584 | 0.7901 | 5.7778 |
|  | Maximum Primer | ESGA-ML-3 | ESGA-ML-3 | ESGA-ML-1/2/3/5/8/9/12/14/16/17/18/19/20 | ESGA-ML-17 | ESGA-ML-20 | ESGA-ML-17 | ESGA-ML-20 |
|  | Mean | 5.75 | 5.25 | 90.52% | 0.4059 | 2.1131 | 0.6494 | 3.3890 |
| ESGS | Total | 118 | 97 | / | / | 36.2146 | 8.7306 | 56.7407 |
|  | Minimum | 4 | 3 | 42.86% | 0.2261 | 1.0755 | 0.2667 | 1.3333 |
|  | Minimum Primer | ESGS-98 | ESGS-41 | ESGS-41 | ESGS-193 | ESGS-41 | ESGS-193 | ESGS-193 |
|  | Maximum | 12 | 12 | 100.00% | 0.4481 | 4.4388 | 0.7654 | 6.8148 |
|  | Maximum Primer | ESGS-87 | ESGS-87 | ESGS-31/87/98/170 | ESGS-170 | ESGS-87 | ESGS-170 | ESGS-87 |
|  | Mean | 7.87 | 6.47 | 82.49% | 0.3716 | 2.4143 | 0.5820 | 3.7827 |
| ES | Total | 91 | 67 | / | / | 22.2217 | 6.8938 | 32.2223 |
|  | Minimum | 3 | 2 | 50.00% | 0.2524 | 0.5048 | 0.2963 | 0.5926 |
|  | Minimum Primer | ES-90 | ES-90 | ES-120 | ES-5/28/90 | ES-90 | ES-90 | ES-90 |
|  | Maximum | 10 | 8 | 100.00% | 0.4027 | 2.8368 | 0.6370 | 4.3703 |
|  | Maximum Primer | ES-24 | ES-24 | ES-85/103 | ES-158 | ES-24 | ES-158 | ES-24 |
|  | Mean | 6.07 | 4.47 | 73.84% | 0.3214 | 1.4814 | 0.4596 | 2.1482 |

TNB, total number of bands; NPB, number of polymorphic bands; PP, percentage of polymorphic bands; PIC, polymorphism information content; MI, marker index; BI, band informativeness; Rp, resolving power.
