# Supplementary material for: Genomic survey sequencing, development and characterization of single- and multi-locus genomic SSR markers of Elymus sibiricus L
Source: BMC Plant Biol. 2021 Jan 6;21:3. doi: 10.1186/s12870-020-02770-0 (PMC7789342; doi:10.1186/s12870-020-02770-0)
Supplement: Supplementary file 8 — Additional file 8: Table S8. Statistical significance of Mann-Whitney test among PIC, MI, BI and Rp of different markers [file 12870_2020_2770_MOESM8_ESM.docx]

**Table S8** Statistical significance of Mann-Whitney test among PIC, MI, BI and Rp of different markers.

| PIC | | | | | MI | | | BI | | | Rp | | |
| --- | --- | --- | --- | --- | --- | --- | --- | --- | --- | --- | --- | --- | --- |
|  | ESGA-SL | ESGA-ML | ESGS | ES | ESGA-ML | ESGS | ES | ESGA-ML | ESGS | ES | ESGA-ML | ESGS | ES |
| ESGA-SL | / |  |  |  |  |  |  |  |  |  |  |  |  |
| ESGA-ML | 0.356 | / |  |  | / |  |  | / |  |  | / |  |  |
| ESGS | 0.222 | 0.036* | / |  | 0.474 | / |  | 0.069 | / |  | 0.559 | / |  |
| ES | 0.134 | 0.001** | 0.012* | / | 0.009** | 0.007** | / | 0.001** | 0.004** | / | 0.004** | 0.004** | / |

PIC, polymorphism information content. MI, marker index. BI, band informativeness. Rp, resolving power. ^*^, *P* < 0.05. ^**^, *P* < 0.01. The ESGS and ES primers were all multi loci markers.
