# Supplementary figures and images for: Genomic survey sequencing, development and characterization of single- and multi-locus genomic SSR markers of Elymus sibiricus L
Source: BMC Plant Biol. 2021 Jan 6;21:3. doi: 10.1186/s12870-020-02770-0 (PMC7789342; doi:10.1186/s12870-020-02770-0)

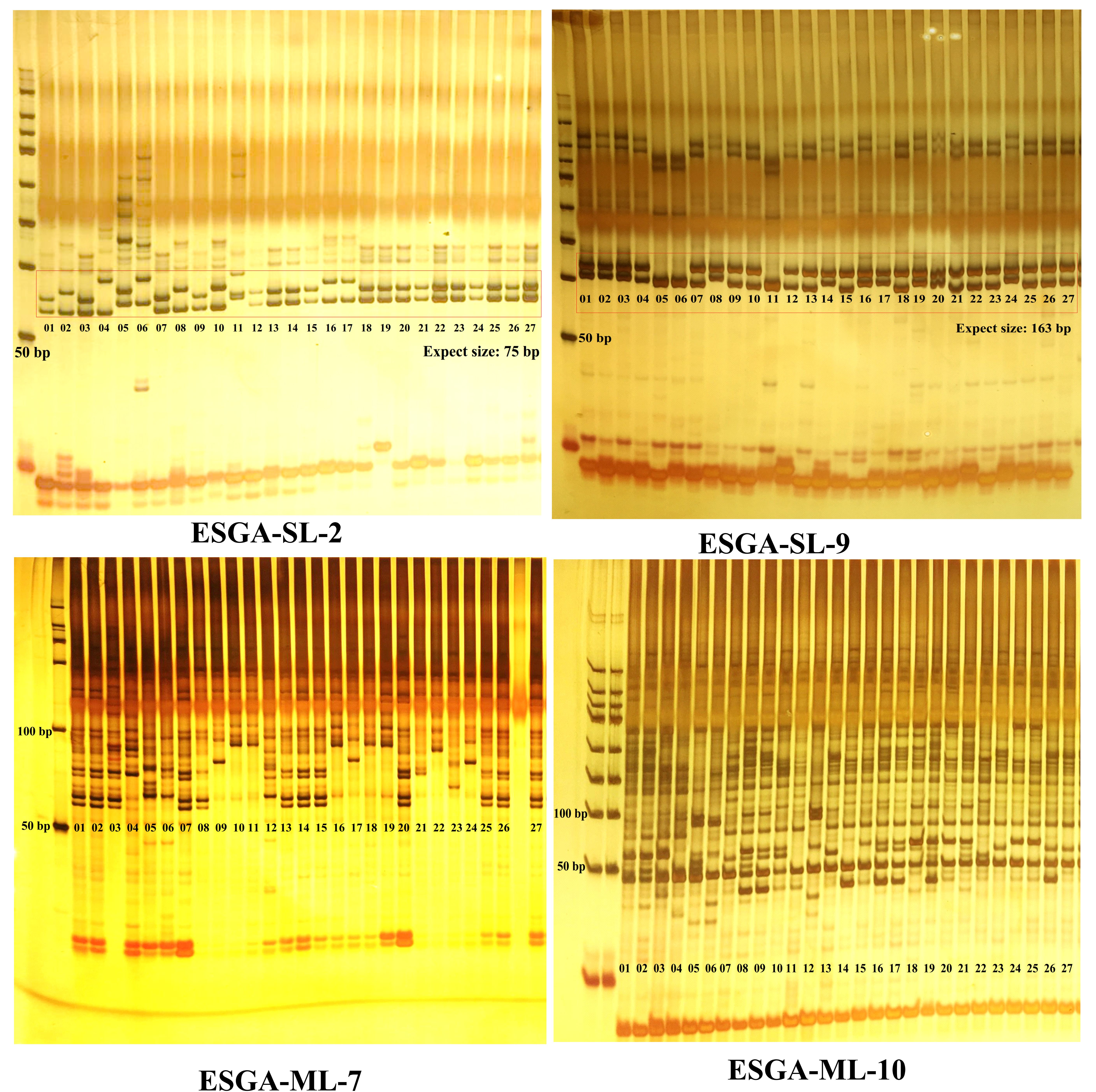

Supplement: Supplementary file 9 — Additional file 9: Figure S1. Example of PAGE electrophoretic picture of single-loci (ESGA-SL-2 and ESGA-SL-9) and multi-locus (ESGA-ML-7 and ESGA-ML-10) markers in this study. [file 12870_2020_2770_MOESM9_ESM.png]

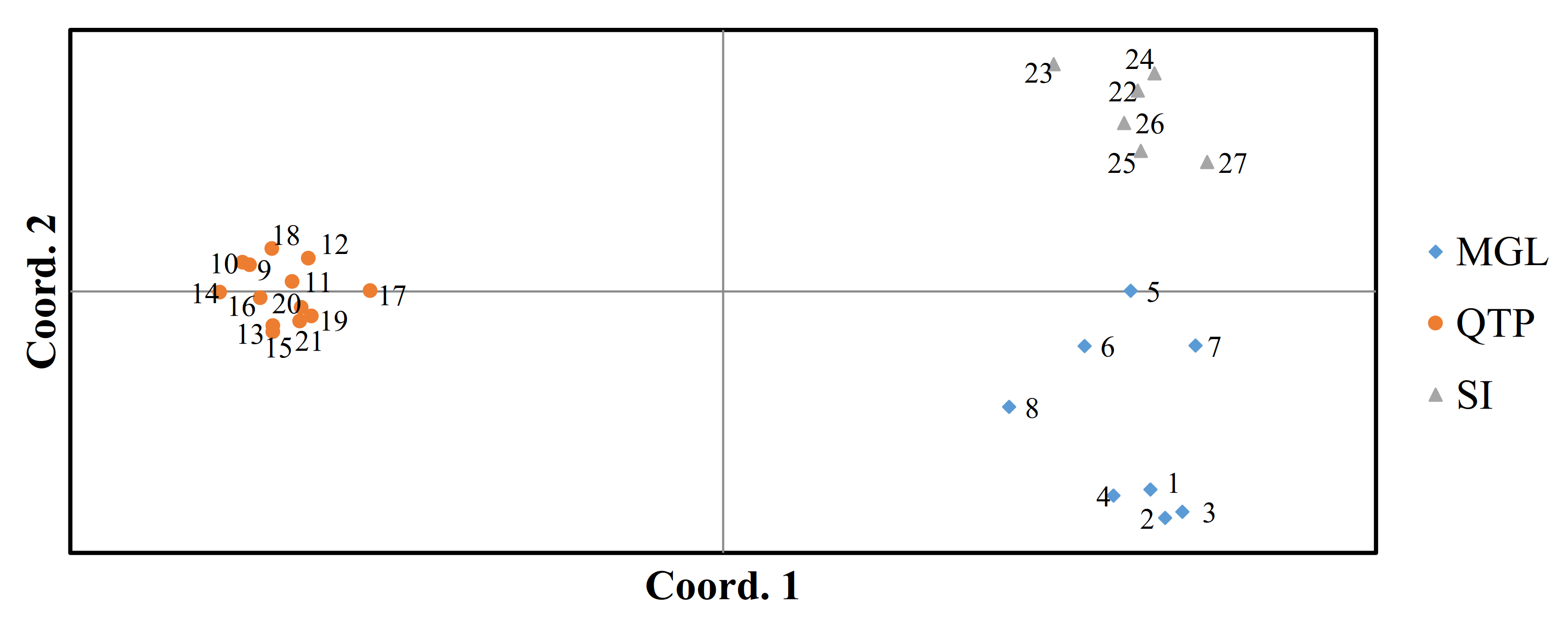

Supplement: Supplementary file 10 — Additional file 10: Figure S2. The UPGMA dendrogram and genetic structure of 27 studied E. sibiricus based on ESGA-ML markers. [file 12870_2020_2770_MOESM10_ESM.tif]

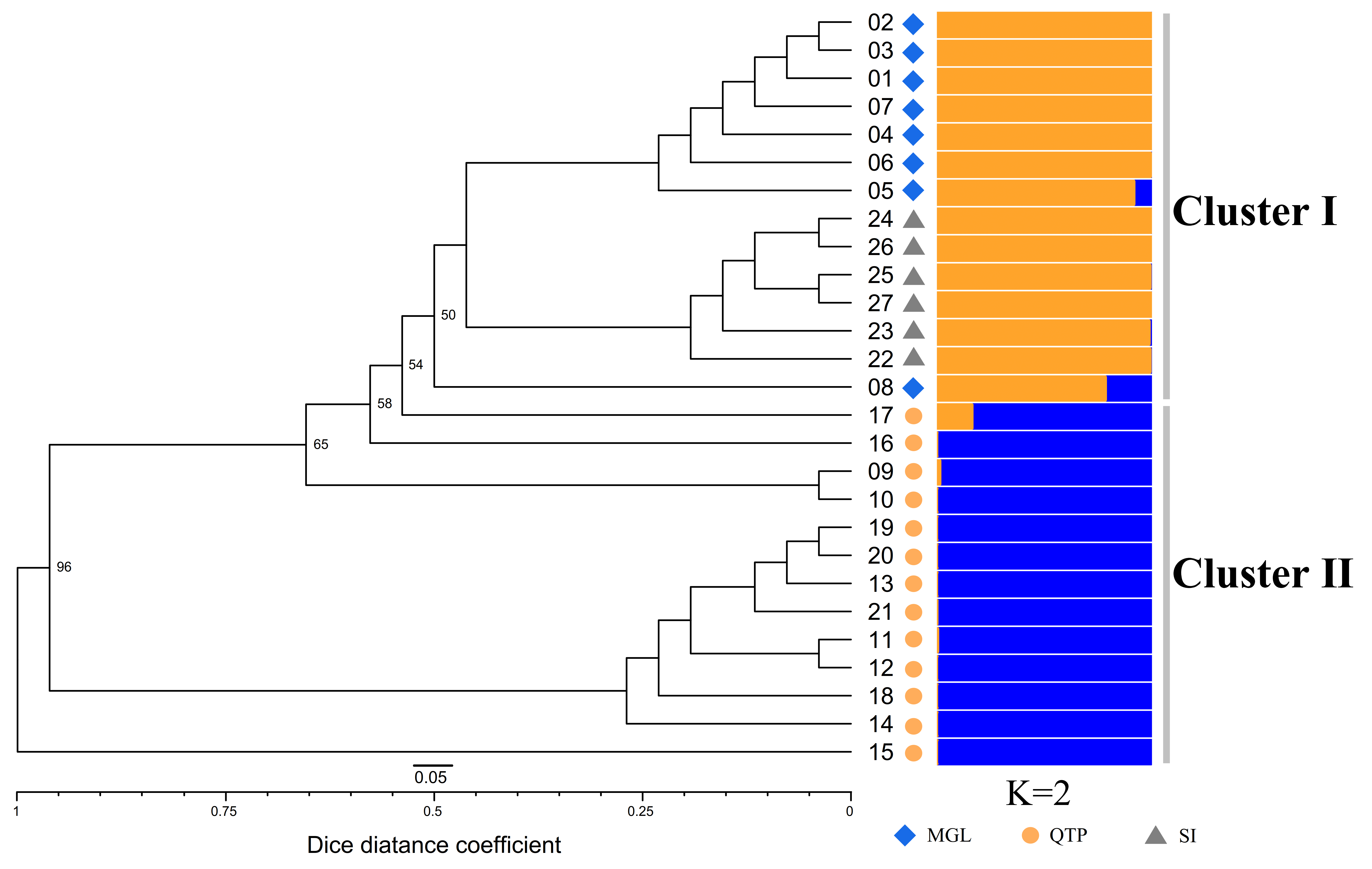

Supplement: Supplementary file 11 — Additional file 11: Figure S3. The PcoA analysis of 27 studied E. sibiricus based on ESGA-ML markers. [file 12870_2020_2770_MOESM11_ESM.tif]

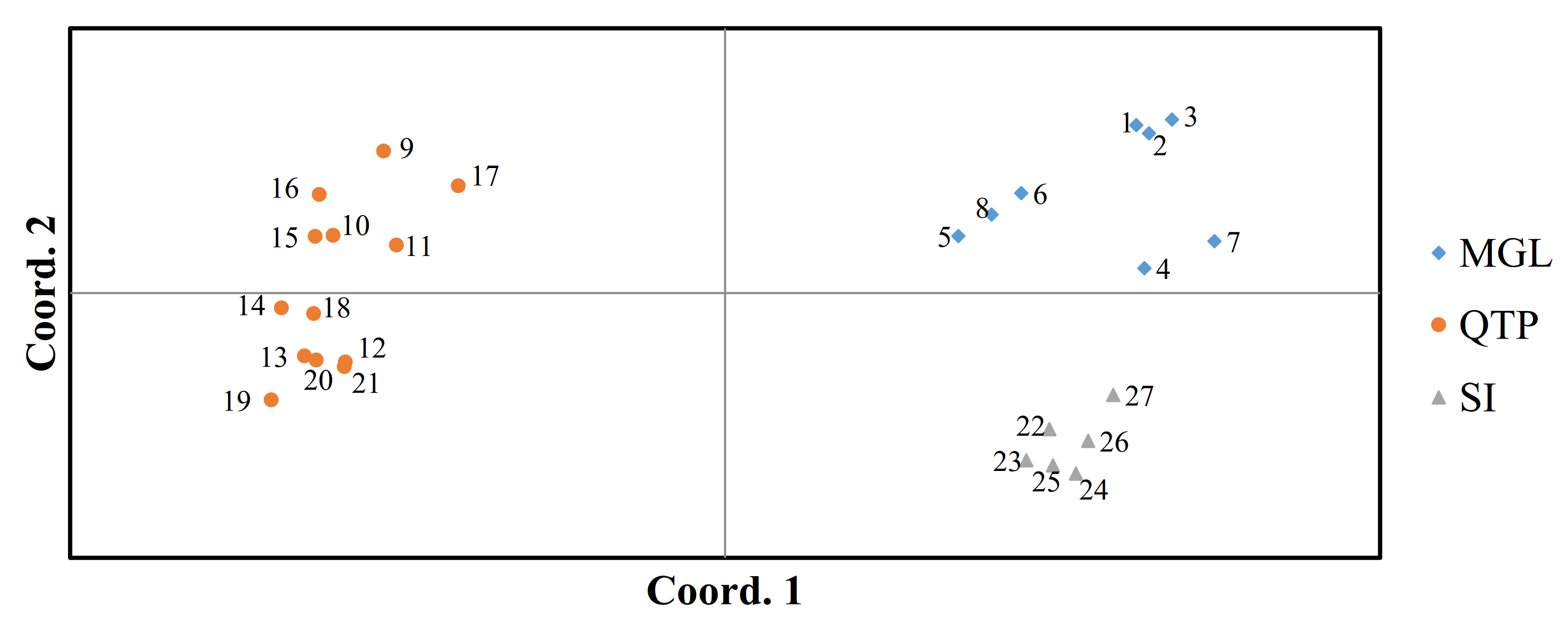

Supplement: Supplementary file 12 — Additional file 12: Figure S4. The UPGMA dendrogram and genetic structure of 27 studied E. sibiricus based on ESGS markers. [file 12870_2020_2770_MOESM12_ESM.tif]

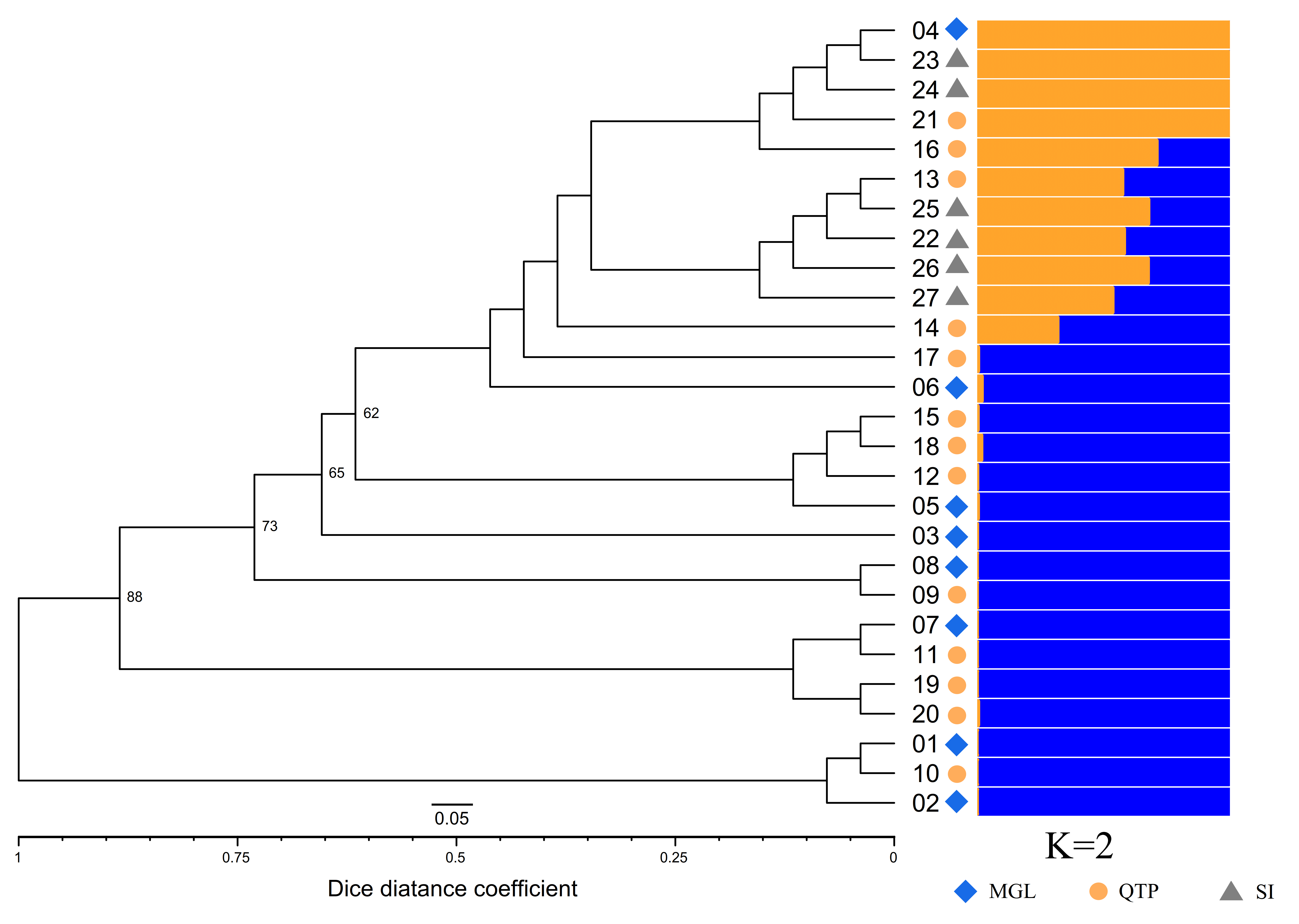

Supplement: Supplementary file 13 — Additional file 13: Figure S5. The PcoA analysis of 27 studied E. sibiricus based on ESGS markers. [file 12870_2020_2770_MOESM13_ESM.tif]

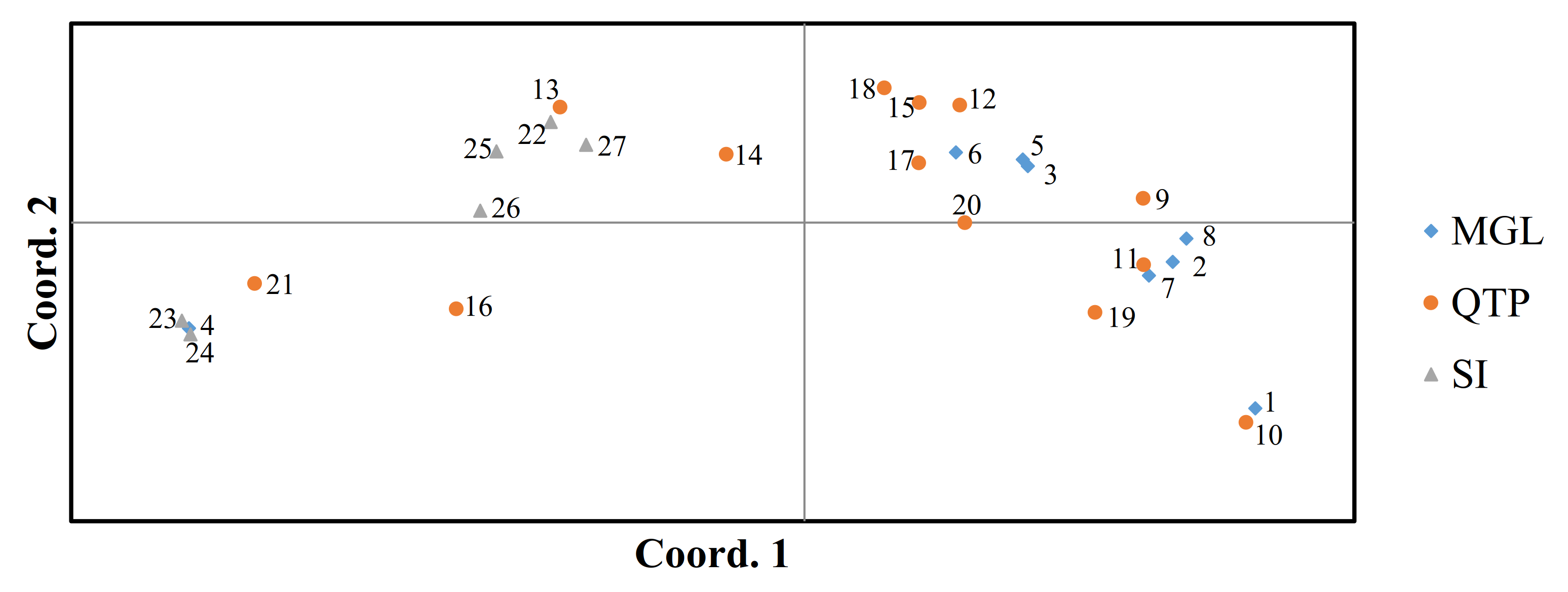

Supplement: Supplementary file 14 — Additional file 14: Figure S6. The UPGMA dendrogram and genetic structure of 27 studied E. sibiricus based on ES markers. [file 12870_2020_2770_MOESM14_ESM.tif]

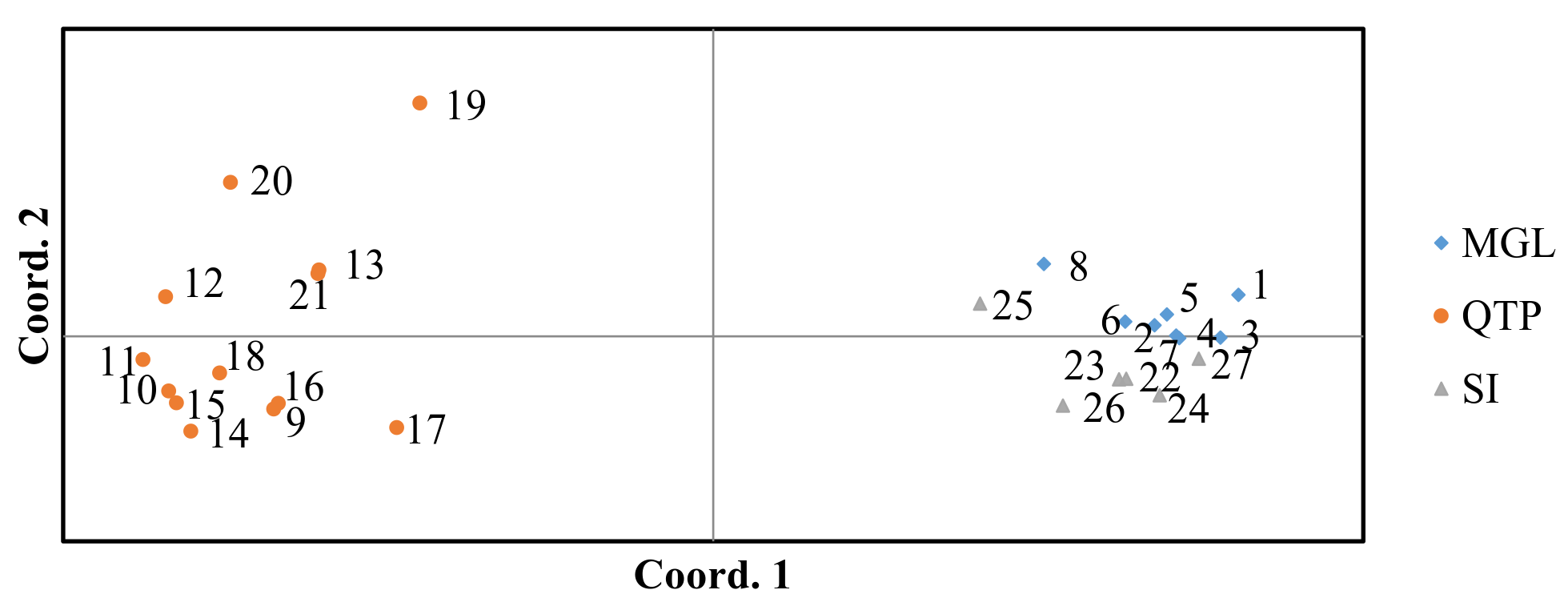

Supplement: Supplementary file 15 — Additional file 15: Figure S7. The PcoA analysis of 27 studied E. sibiricus based on ES markers. [file 12870_2020_2770_MOESM15_ESM.tif]

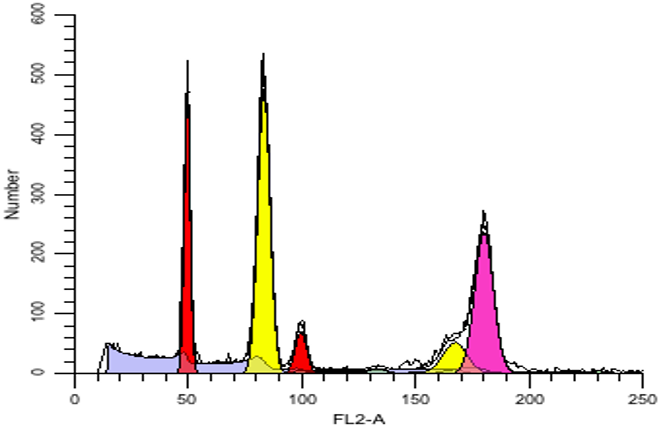

Supplement: Supplementary file 16 — Additional file 16: Figure S8. E. sibiricus cultivar ‘Chuancao No.2’ was identified as tetraploid by flow cytometry. Red color is diploid Hordeum vulgare L., yellow is E. sibiricus cultivar ‘Chuancao No.2’ and pink is hexaploid Triticum aestivum. [file 12870_2020_2770_MOESM16_ESM.png]
